# Comparing XAI techniques for interpreting short-term burglary predictions at micro-places

**DOI:** 10.1007/s43762-025-00185-x

**Published:** 2025-05-09

**Authors:** Robin Khalfa, Naomi Theinert, Wim Hardyns

**Affiliations:** 1 Department of Criminology, Criminal Law and Social Law, Ghent University, Universiteitstraat 4, Ghent, 9000 Belgium; 2 Faculty of Social Sciences, University of Antwerp, Sint-Jacobstraat 2, Antwerp, 2000 Belgium

**Keywords:** Crime prediction, Crime and place, Machine learning, Explainable AI, Micro-place, XGBoost, Burglary

## Abstract

This study empirically compares multiple eXplainable Artificial Intelligence (XAI) techniques to interpret short-term (weekly) machine learning-based burglary predictions at the micro-place level in Ghent, Belgium. While previous research predominantly relies on SHAP to interpret spatiotemporal crime predictions, this is the first study to systematically evaluate SHAP alongside other XAI techniques, offering both global and local model interpretability within the context of crime prediction. Using data from 2014 to 2018 on residential burglary, repeat and near-repeat victimization, environmental features, socio-demographic indicators, and seasonal effects, we trained an XGBoost model with 76 features to predict weekly burglary hot spots. This model serves as a basis for comparing the interpretative power of different XAI techniques. Our results show that built environment and land use characteristics are the most consistent global predictors of burglary risk. However, their influence varies substantially at the local level, revealing the importance of spatial context. While global feature importance rankings are broadly aligned across XAI techniques, local explanations, especially between SHAP and LIME, often diverge. These discrepancies highlight the need for careful method selection when translating predictions into crime prevention strategies. In addition, this study demonstrates that short-term burglary risks are influenced by complex interactions and threshold effects between environmental and social disorganization features. We interpret these findings through the lens of criminological theory, and argue for more integrated approaches that go beyond examining the isolated effects of specific crime predictors. Finally, we call for greater attention to the methodological implications that arise from applying different interpretability techniques, particularly when machine learning model outputs are used to inform crime prevention and policy decisions.

**Supplementary Information:**

The online version contains supplementary material available at 10.1007/s43762-025-00185-x.

## Introduction and background

### The use of machine learning to predict the risk of crime at micro-places

Recent advances in environmental criminology and the criminology of place increasingly highlight the potential of machine learning methods for predicting where a crime is likely to occur in the (near) future (Caplan & Kennedy, 2016; Chainey et al., 2008; Mohler et al., 2011; Rosser et al., 2017; Rummens & Hardyns, 2020). Compared to traditional statistical methods used to predict crime at micro-places, such as Risk Terrain Modeling, which relies on conventional regression techniques, or near repeat models, machine learning methods are agued to provide a more versatile toolkit for modeling the risk of crime at places over both short- and long-term periods (Rummens & Hardyns, 2020; Wheeler & Steenbeek, 2021). This is especially true for nonparametric machine learning methods, such as neural networks or ensemble methods including the random forest or gradient boosting machines, which offer greater flexibility for modelling non-linear effects and relationships in high-dimensional settings, i.e. when a large number of features and observations are involved and when numerous interactions between features are assumed (James et al., 2013). This flexibility proves increasingly valuable, given not only the growing complexity and volumes of fine-grained spatiotemporal data that can be used for the purpose of predicting spatiotemporal crime patterns (Snaphaan & Hardyns, 2021), but also the wide range of factors and interactions between factors that have been proposed as potentially predictive of crime at specific places and times (for a broader discussion, see Wheeler & Steenbeek, 2021).

The latter is particularly evident in the predictive modeling of street crimes, such as residential and commercial burglary, street robbery, and assault, at the micro-place level. Drawing on crime opportunity theories, including routine activity (Cohen & Felson, 1979) and crime pattern theory (Brantingham & Brantingham, 1993a, 1993b), for example, prior research has identified a wide range of environmental and situational factors as potential predictors of street crime. These typically relate to the environmental backcloth of criminal opportunities and the convergence of people in space and time (Wheeler & Steenbeek, 2021). Common predictors include the density of or proximity to specific crime generators (e.g., bars, liquor stores, ATMs) (e.g., Haberman & Ratcliffe, 2015; Kubrin & Hipp, 2016; Wheeler, 2019), crime attractors (places that attract motivated offenders due to specific opportunities) (e.g., Bernasco & Block, 2011) and crime detractors (e.g., police stations, entry controls, or CCTV surveillance) (e.g., Kinney et al., 2008; Piza et al., 2019; Wang et al., 2021). Other features of the built environment, including street layout, land use diversity, lighting, visibility, and accessibility, have also been linked to variations in street crime risks (e.g., Perkins et al., 1993; Frith et al., 2017; Xie et al., 2022; Yue et al., 2023) . Furthermore, the use of ambient population estimates, which are increasingly derived from big data sources such as social media and mobile phone data, has also been shown to improve street crime predictions by better capturing human mobility and the dynamic convergence of offenders, targets, and guardianship, particularly for crimes involving mobile targets (e.g., Rummens et al., 2021). Also, studies have demonstrated the role of time-dependent factors, including seasonality, weather, and (near) repeat victimization, in shaping street crime risks (Andresen & Malleson, 2013; Corcoran & Zahnow, 2022; Pease, 1998). Additionally, while much micro-place-based research has focused on the influence of crime opportunities and the physical environment, there is growing recognition of the need to integrate neighborhood-level theories, such as social disorganization theory, to account for potential interactions between structural area characteristic and crime opportunity structures (e.g., Jones & Pridemore, 2019; Weisburd, 2015).

Hence, as the complexity of potential crime predictors and their interactions increases, nonparametric machine learning methods stand out for their ability to handle these complexities with fewer statistical assumptions, such as linearity and multicollinearity (Kim & Lee, 2023). This capability has been shown to reduce overfitting, achieving a better balance between model bias and variance, thereby improving model performance and generalizability (James et al., 2013). For example, Rummens et al. (2017) found that an ensemble machine learning model combining a multi-layer perceptron neural network and logistic regression outperformed near-repeat and risk terrain models in predicting residential burglary hotspots in Belgium. Similarly, Wheeler and Steenbeek (2021) demonstrated that a random forest model outperformed a risk terrain model and kernel density estimation in predicting long-term street robberies in Dallas. Zhang et al. (2022) also showed that an XGBoost model outperformed simpler models like logistic regression and decision trees in predicting public street theft.

### Interpreting machine learning-based crime predictions

Although studies have shown that nonparametric machine learning methods can model non-linearities, capture complex feature interactions, and improve (street) crime prediction performance, these gains often come at the cost of interpretability and, ultimately, explainability (Wheeler & Steenbeek, 2021; Zhang et al., 2022, 2023). As a result, most nonparametric machine learning method are frequently criticized for their ‘black box’ nature (Kim & Lee, 2023; Wheeler & Steenbeek, 2021). This implies that it is generally difficult to determine which features influence the predictions, in what way features and interactions between features influence the predictions and why a model predicts specific instances at a more local level (James et al., 2013).

The ability to interpret machine learning model outputs and understand the processes shaping their predictions is not only a technical concern but also a critical requirement for end users, including police officers, policymakers, and the public, as well as a legal obligation (Laato et al., 2022; Vestby & Vestby, 2021). When predictions cannot interpreted in a way that is accessible to users, the practical utility of the model is undermined (Stiglic et al., 2020). This may result in models that fail to meet operational demands and usability standards, ultimately limiting their adoption. Beyond regulatory compliance, interpretability is also essential for public trust and for identifying and mitigating potential biases or errors in predictive models (Amarasinghe et al., 2023). These concerns highlight the need for methodologies that enable the interpretation of model predictions, which has led to growing interest in the field of Explainable Artificial Intelligence (XAI) (Mehta et al., 2023). The core aim of XAI is to ‘white-box’ machine learning models by making their internal decision processes more accessible and understandable to humans (Molnar, 2020). This is particularly relevant in criminological applications, where the use of ‘black-box’ models to predict crime risk raises questions of transparency, trust, and accountability.

Several studies have applied XAI techniques to improve the interpretability of machine learning-based crime predictions. Wheeler and Steenbeek (2021), for example, employed permutation-based feature importance test, Accumulated Local Effect (ALE) plots, and Shapley Additive Explanations (SHAP) to interpret random forest predictions of long-term robbery risk in Dallas, illustrating the potential of these tools to support localized, problem-oriented policing. Similarly, Xie et al. (2022) applied SHAP to interpret street crime predictions in Zigong, China, highlighting important interactions between streetscape features and street crime risk. Kim and Lee (2023) also demonstrated SHAP’s ability to capture non-linear relationships and local feature interactions, including the (nonlinear) influence of bars, restaurants, and CCTV cameras, in crime predictions for Seoul, South Korea. Other contributions include Zhang et al. (2023) and Deng et al. (2023), who applied SHAP to long-term crime risk models in Beijing and Dallas, confirming its value for revealing complex spatial associations at varying grid scales. Another notable contribution is the work of Zhang et al. (2022), who pioneered the application of SHAP to short-term crime predictions in Zigong city (China), focusing on interpreting biweekly public theft predictions at a 150-m grid resolution.

Despite these prior contributions, several important gaps remain. First, most studies rely on SHAP to provide both global and local explanations for machine learning-based crime predictions, with limited comparison to alternative XAI techniques (e.g., Agarwal & Das, 2020). While SHAP is widely recognized for its flexibility, evaluating whether other methods produce consistent or divergent interpretations is essential both for theoretical development and for informing the appropriate selection of interpretability tools in applied contexts. Second, prior research has primarily focused on long-term crime predictions, with only Zhang et al. (2022) addressing the interpretation of short-term predictions. Given that micro-geographic crime patterns often fluctuate over short time periods, the ability to generate and interpret short-term crime predictions (e.g., daily, weekly, or monthly predictions) may prove valuable for operational decision-making and resource allocation in response to emerging crime trends and problems. Third, existing studies are largely concentrated in North America and East Asia, leaving European settings underexplored. Since spatiotemporal crime dynamics and their correlates are context-dependent, expanding research to European cities is essential to assess the generalizability of current findings and to inform both theory and practice.

### The present study

This study aims to address these existing gaps by empirically evaluating and comparing various XAI techniques to guide both global and local interpretations of weekly micro-geographic residential burglary predictions derived through applying a nonparametric machine learning model, using the city of Ghent (Belgium) as a case study.​ The choice to solely focus on residential burglary as crime type is due to the high spatial accuracy of the crime data, clear spatiotemporal concentration, and the advantage of conducting an in-depth analysis on a single crime type. In addition, the city of Ghent, as a mid-sized European city, offers a valuable context to extend the literature on interpreting machine learning-based crime predictions beyond large metropolitan areas.​

Our analysis explores three distinct levels of interpretability:

1. *Global feature importance:* First, we assess which features are most influential in the overall model predictions by comparing different feature importance tests. Prior studies have mainly used SHAP-based feature importances to identify key predictors of long-term spatial crime patterns (e.g., Wheeler & Steenbeek, 2021), but few have evaluated the consistency of results across different feature importance tests, particularly in the context of short-term crime predictions. The present study therefore assesses to what extent different feature importance test converge on similar conclusions and offers insights into their methodological reliability.
2. *Global feature effects and interactions:* Second, we examine how specific features influence model predictions, focusing on exploring both the direction and magnitude of feature effects, as well as potential (nonlinear) interactions between features. Mapping these effects and interactions is especially relevant for complex crimes such as residential burglary, where the combination of features often shapes risk patterns in unexpected ways. While SHAP has mainly been used for this purpose, we compare and assess the robustness of SHAP alongside other methods in identifying feature effects and interactions and evaluate the consistency of the insights derived.
3. *Local interpretability:* Third, we investigate specific burglary predictions for particular locations and time periods to understand why a specific prediction occurs, identifying which features contribute to a specific predicted outcome. While prior studies have primarily relied on SHAP for local interpretation, this study compares whether different methods produce consistent explanations for the same predicted instances (individual predictions).

### Data

This study is set in Ghent, Belgium, an urban area of 156 km^2^ encompassing 261,475 inhabitants (based on 2018 data). The selection of the data sources and features used in the machine learning modelling is grounded in criminological theories, including (near) repeat victimization, crime pattern theory, routine activity theory, social disorganization theory, and recent empirical work on predictive (street) crime modelling (e.g., Hardyns & Khalfa, 2023; Kadar et al., 2019; Wheeler & Steenbeek, 2021; Zhang et al., 2022, 2023). A summary of all data sources and features used is available in Appendix A (in the supplementary material).

### Police-registered data

Police-registered residential burglary records spanning the period 2014–2018 were obtained from the Local Police of Ghent, detailing the dates, times, and place indications (raw addresses) of residential burglary events. Following established methodologies in crime prediction research, the raw addresses were geocoded and the data were aggregated to a 200 by 200-m grid, resulting in 4,258 cells for the city of Ghent.^1^ In addition, the midpoints of the date-time ranges were used when exact timestamps were unavailable, excluding events with time ranges exceeding seven days, leaving 8,049 burglary events for further analysis. The data were subsequently aggregated into weekly windows to develop a prediction dataset where each observation represents a unique grid cell-week combination. A total of 20 crime history features were constructed to capture repeat and near-repeat victimization patterns. Repeat victimization refers to the increased likelihood of a location being targeted again after it has already been victimized (Bernasco et al., 2015; Johnson et al., 2009; Pease, 1998), while near-repeat victimization extends this assumption to the surrounding area, suggesting that locations in close proximity to a previously victimized area are also at a heightened risk of being targeted (Bowers & Johnson, 2004; Townsley et al., 2003). Both repeat and near-repeat victimization patterns have been shown to influence residential burglary risks, with prior incidents at a specific location or in its vicinity increasing the risk of future burglaries.

### Environmental characteristics

Data on various Points of Interest (POIs) and land use characteristics were furthermore added to the prediction dataset to account for the potential influence of the built environment as well as crime generators, attractors, and detractors on burglary risk. These data sources were drawn from three key open data repositories, namely OpenStreetMap, the open data repository of the City of Ghent, and the open data repository of the Flemish Government of Belgium. Most POI indicators were available at the point level and were collected individually to capture their distinct impact on the predicted crime patterns. In some cases, however, POIs were aggregated into broader categories (such as shops, public buildings, restaurants and cafés, and nightclubs and bars). For 8 POI indicators, we opted to model their impact via both a density and a distance function (Wheeler & Steenbeek, 2021). Hence, two distinct features were created for these POI indicators: one representing the density of each POI in each grid cell, calculated using a normal kernel density function, and another representing the distance from each grid cell to the nearest POI. For 11 additional POI indicators, we opted to use only the distance to the nearest POI as a feature as there were too few data points to create meaningful density measures. The land use data furthermore consisted of geo-located polygons classified into 17 distinct categories, which were subsequently consolidated into 10 features representing proportional land usage per grid cell. Finally, two urban form indicators were added to the model: 1) a street connectivity score, measuring how well streets are (inter)connected to each other in a grid cell, and 2) a function mix score, quantifying the diversity of functions (e.g., offices, shops, sports facilities, green spaces, schools, and hospitals) within a grid cell.^2^

### Socio-demographic and -economic characteristics

The included socio-demographic and economic data were collected via the open data repository of the Belgian national government. These data were used to develop features aimed at modeling the impact of structural area characteristics on spatial crime patterns as proposed by social disorganization theory (Sampson, 1985; Sampson & Groves, 1989; Sampson et al., 1997; Shaw & McKay, 1969). The socio-demographic and economic data were only available at the neighborhood level, however, which is why we disaggregated the neighborhood-level data to the grid level. This disaggregation was done using a simple areal weighted interpolation method, allowing for the redistribution of data from larger geographic units to smaller ones based on spatial proportional allocation.^3^

### Seasonality and weather conditions

Finally, several features are included that relate to seasonality and weather conditions. These features are included in order to account for the potential varying effects of seasonality and weather conditions on spatial crime patterns. Prior research suggests that seasonality and weather conditions can affect crime through changes in routine activities and environmental conditions, potentially influencing both the opportunities for crime and the behavior of individuals (see Corcoran & Zahnow, 2022 for a systematic overview). For example, certain crimes might increase during specific seasons due to changes in social behavior or environmental factors (e.g., increased social gatherings during warmer months). Similarly, bad weather conditions might reduce outdoor activities and thereby lower the opportunities for certain types of crime such as residential burglary, while favorable weather might have the opposite effect.

## Methods

### Machine learning modeling

The final prediction dataset consists of 76 features aggregated over 4258 grid cells and 260 weekly cycles (5 years with each 52 weeks), totaling 1,107,080 spatiotemporal observations. To ensure the robustness of the modeling and to address potential privacy concerns associated with sparse spatiotemporal data, grid cells with less than 5% residential area of the total cell area are excluded from the analysis, resulting in 483,430 spatiotemporal observations (1859 grid cells × 260 weekly cycles). The dataset is split into 4/5 training data (2014–2017) and 1/5 test data (2018) in order to predict weekly residential burglary hot spots throughout 2018. The prediction of residential burglary hot spots is done through a binary classification task, estimating the probability of one or more burglaries occurring within a grid cell over a one-week period.^4^ This approach is typically referred to as a ‘ranking task’ in machine learning, where the focus is on ranking observations according to their predicted probabilities.

The eXtreme Gradient Boosting (XGBoost) algorithm is selected to derive the burglary predictions.^5^ XGBoost is a powerful and widely recognized machine learning algorithm for modeling structured, tabular data (James et al., 2013) and has already been successfully applied within the context of spatiotemporal crime prediction (e.g., Xie et al., 2022; Zhang et al., 2022, 2023). Unlike bagging methods (such as random forests), which train trees independently on random subsets of the data and subsequently aggregate their predictions, XGBoost is a boosting algorithm that follows a sequential approach in building decision trees (Chen & Guestrin, 2016). In this regard, each new tree is fitted to the residuals (the errors made by the previous decision tree in the ensemble) allowing to adjust for potential misclassifications (James et al., 2013; Zhang et al., 2022). One major advantage of XGBoost in this regard is that it allows to control model complexity and reduce the risk of overfitting, a common issue in machine learning where the model becomes too closely tailored to the training data and performs poorly on unseen data (see Chen & Guestrin, 2016 for adetailed description of the XGBoost algorithm). As only 1.51% of all spatiotemporal observations in the training data are labeled as a burglary event, however, the XGBoost model is trained on a resampled version of the training dataset using a five-fold cross-validation scheme to tune the hyperparameters and regularize the training process.^6^ This is necessary to mitigate the bias of the model towards predicting the majority class (i.e., no burglary) (James et al., 2013).

### Model evaluation

Although evaluating the predictive performance of the model is not the primary objective of this study, we present two specific performance metrics to provide a general overview of the performance of the machine learning model in predicting weekly residential burglary hot spots in the city of Ghent.

First, we report the average weekly direct hit rate, referring to the proportion of correctly predicted crime hot spots relative to the actual number of places where one or more criminal events occurred (Rosser et al., 2017). To represent the model’s hit rate, we use a surveillance plot, which visualizes the hit rate (y-axis) as a function of increasing coverage area (x-axis) (Rosser et al., 2017). This plot generally provides an effective tool to illustrate prediction accuracy: monitoring the top x highest-risk areas would capture approximately y of all crimes. In this regard, surveillance plots inherently reflect the so-called Prediction Accuracy Index (PAI), which is defined as the hit rate divided by the coverage area. While our application focuses on visualizing model performance, surveillance plots can also be linked to other types of spatial crime analysis, such as semi-variogram models that quantify spatial dependencies and autocorrelation in crime patterns (e.g., Yu et al., 2020).

Second, we report the Receiver Operating Characteristic (ROC) curve(s) and the average Area Under the Curve (AUC) of the ROC plot(s). In general, the ROC visually presents the performance of a classifier by plotting the true positive rate (or the sensitivity of the classifier) against the false positive rate. By examining the trade-off between true and false positive rates, the ROC curve provides an overview of the capability of a model to correctly classify the instances (James et al., 2013). In this regard, the AUC is a specific summary metric that is derived from the ROC plot, which represents the likelihood that a classifier will rank a randomly chosen positive instance higher than a randomly chosen negative one. An AUC value of 1 indicates perfect classification, while an AUC of 0.5 reflects random performance.

### Model interpretability

We employ and compare various XAI techniques to assess machine learning model interpretability at three specific levels, namely global feature importance, global feature effects and interactions and local interpretability. Rather than relying on one specific technique, we evaluate how SHAP and comparable techniques contribute to interpreting model predictions at these three specific levels.

#### Global feature importance

The first level focuses on identifying the global importance of features used in the machine learning model for predicting burglary hot spots. Three complementary techniques are applied here.

First, as a baseline method, we compute feature importances derived from applying the XGBoost algorithm to the training data. This model-specific approach derives feature importance scores based on how frequently features are used to make decisions within the ensemble of decision trees ( Zheng et al., 2017). In this study, feature importances are calculated using the ‘gain’ metric, which measures the average value of the information gain that is obtained when a feature is used to split a (decision) node in a decision tree (referred to as ‘impurity importance’). It measures how much a specific feature improves model performance by reducing the node impurity (error) at each split. This value is then averaged across all splits and trees in the ensemble to obtain an indication of the overall contribution of a feature.

Second, we compute permutation-based feature importances. Unlike model-specific approaches, permutation-based feature importances are model-agnostic and are typically computed on a separate hold-out test dataset. Permutation-based feature importances provide a more honest measurement of feature importances by randomly ‘permuting’ the values of a feature, thus disrupting/breaking the relationship between the feature and the target variable and measuring the resulting decrease in model loss on the test dataset caused by this permutation (Molnar, 2020; Wheeler & Steenbeek, 2021). In this study, permutation-based feature importances are calculated by measuring the decrease in AUC values after randomly permuting each feature five times. Importantly, permutation-based feature importance implicitly accounts for interaction effects, since the impact of permuting a single feature depends not only on its individual contribution but also on how it interacts with other features in the model.

Lastly, we compute mean SHAP values. In general, SHAP offers a post-hoc model-agnostic approach to measuring feature importance, and is particularly useful for understanding which and how individual features contribute to model predictions, both at a global level and at the level of individual predicted instances. In this regard, SHAP is based on coalitional game theory (Shapley, 1953) and extends earlier applications of model interpretability (Datta et al., 2016;Štrumbelj & Kononenko, 2014). The main idea behind SHAP is that each predicted instance is seen as a payout in a cooperative game, where each feature acts as an individual player. The SHAP value for a specific feature thereby represents the marginal contribution of a feature in fairly distributing the payout (i.e., the model’s prediction) among all possible combinations of feature values, inherently capturing both main and interaction effects. SHAP values thereby provide an estimate of how much each feature in a machine learning model contributes to the observed differences between a prediction and the average predicted value by the model. In this study, we employ a tree-based variant of SHAP (TreeSHAP) recommended for use with tree-based models and capable of accounting for feature dependencies (Lundberg & Lee).

#### Feature effects and interactions

The second level examines how specific features affect model predictions, including their direction, magnitude, and (potential) interactions with other features. Two main techniques are employed here.

Accumulated Local Effect (ALE) plots are employed to assess how the features impact the overall machine learning model. Unlike Partial Dependence Plots (PDPs), which also allow to illustrate the marginal effects of features but assume independence between features, ALE plots can handle correlation between features as they average the changes in the predictions and amass over the grid (Apley & Zhu, 2020; Molnar, 2020.). ALEs are estimated by calculating how model predictions change when a specific feature is varied within small intervals, averaging these changes over all observations in the entire distribution. We employ both one- and two-dimensional ALE plots. The one-dimensional plots allow to evaluate the direction and magnitude of the effects of one specific feature on the model predictions, whereas the two-dimensional plots allow to examine potential (second-order) interactions between features.

In addition, one- and two-dimensional SHAP dependence plots are employed as an alternative approach to examine the size and magnitude of feature effects on model predictions and to visually investigate interaction effects between specific features. SHAP dependence plots allow to show the variance in the effects of specific features across all individual predicted instances by measuring the marginal contribution to the prediction compared to all other possible combinations of feature values (Molnar, 2020; Shapley, 1953). In this way, SHAP dependence plots allow to account for and examine potential local heterogeneity of feature effects and interactions.

#### Local interpretability

The third level focuses on investigating why specific predictions are made at a local level. While SHAP excels in providing detailed local and global explanations, it is computationally intensive. Local Interpretable Model-agnostic Explanations (LIME) are therefore used and compared to SHAP. In general, LIME provides detailed local insights learned on perturbations of the original predicted instance. LIME thereby changes the feature values of a single observation to observe how these changes affect the model’s predictions. In this regard, LIME perturbates the original data instances and then uses this new dataset to fit a more interpretable model that approximates the black-box model’s behavior locally around the instance being explained (see Molnar, 2020, for a detailed overview). Since both LIME and SHAP are model-agnostic, they can be applied to a wide range of models and can offer local explanations and comparative explanations across different models. To complement the current understanding of these techniques, it can be further insightful to assess their differences and similarities regarding the explanations they offer and enhancing the understanding for specific predictions and facilitating recommendations.

## Results

### Model performance

**Fig. 1:**
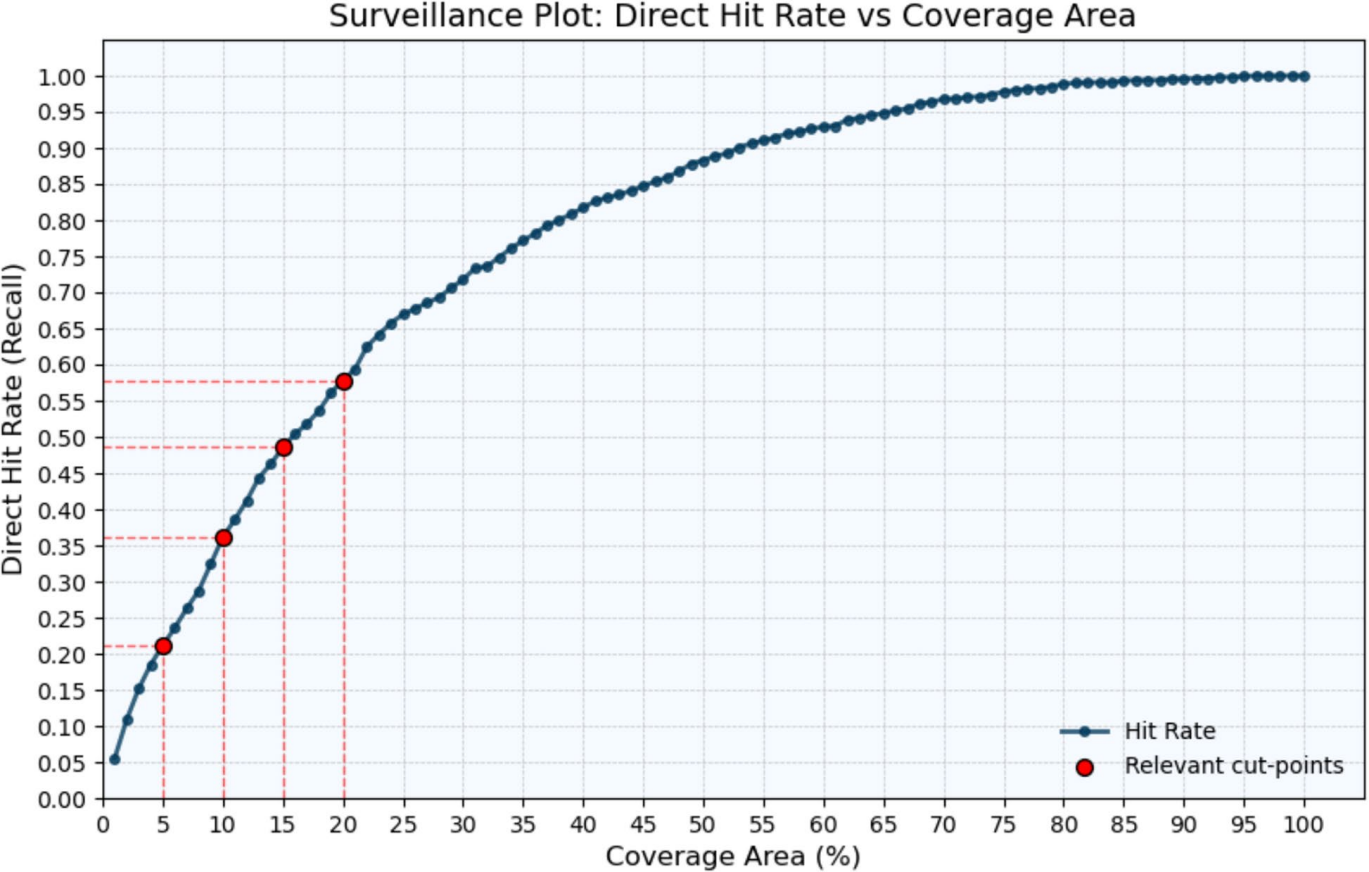
Surveillance plot: Direct hit rates as a function of the percentage of the total study area predicted to be at risk (coverage area)

Figure 1 shows that the XGBoost model is able to capture a meaningful portion of actual burglary hot spots. Notably, when predicting the top 5% of the total study area, the model correctly identifies approximately 21% of the grid cells where one or more residential burglaries occurred. As the predicted area coverage level increases to 10%, 15%, and 20% of the total area the model's performance improves, correctly predicting 36%, 49%, and 58% of the actual burglary locations. This model performance is relatively consistent with results from prior studies that have employed similar spatiotemporal resolutions for crime prediction (e.g., Kadar et al., 2019; Rummens & Hardyns, 2021).

To assist in the interpretation of the direct hit rates, Appendix B (in the supplementary material) presents four spatial maps illustrating the predicted high-risk areas for residential burglary across Ghent in 2018. Each map represents a different predicted area coverage threshold, focusing on the most relevant area coverage area levels: (a) 5%, (b) 10%, (c) 15%, and (d) 20% of the total study area per week. The maps display all unique grid cells that were classified as high-risk at least once. The color gradient indicates how frequently each grid cell was flagged as high-risk, with darker shades representing areas predicted as high-risk for a greater number of weeks. In general, it can be observed that across all presented area coverage levels, the predictions are predominantly concentrated in the city center. In contrast, burglaries occurring in the peripheral areas, particularly in the southwest, are rarely captured by the model. As the coverage threshold increases, more areas are identified as high-risk, with predictions extending outward from the city center toward the northeast and northwest parts of the city. Hence, as the predicted coverage area increases, lower-ranked grid cells with lower predicted probabilities are progressively included in the high-risk category. However, even at the 20% coverage level, predictions remain largely absent in certain peripheral areas where burglaries occurred, suggesting potential blind spots not captured by the model. Increasing the number of predictions per week also introduces less variability in the number of weeks each unique grid cell is predicted as high risk, with areas in the city center being predicted more frequently, while areas on the periphery are predicted only sporadically.

**Fig. 2:**
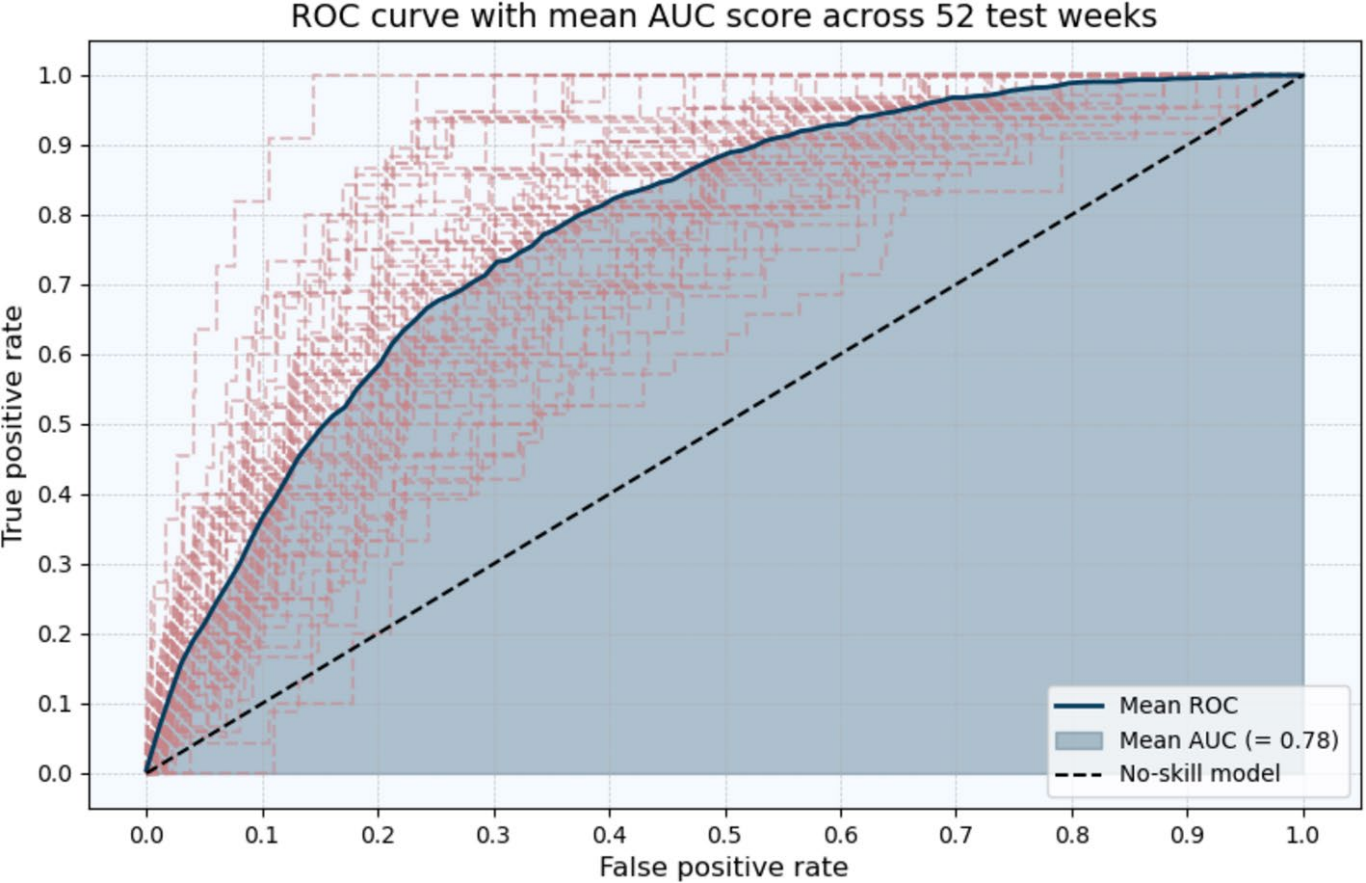
ROC curve(s) and mean AUC score across all 52 weeks in the test dataset

Furthermore, Fig. 2 presents the ROC curves for each individual week in the test dataset (displayed in red) as well as the mean AUC value. The mean AUC value of 0.78 indicates that, on average, the model performs relatively well in distinguishing between areas with a high likelihood of burglary and those with a lower risk. An AUC value of 0.78 signifies that the model is effective at ranking locations based on their predicted risk of burglary, where a value of 1.0 represents perfect discrimination and a value of 0.50 indicates performance equivalent to random chance. The fact that the AUC is significantly higher than 0.50 demonstrates that the XGBoost model outperforms random chance (no discrimination).

### Global feature importances

#### Model-specific feature importances

**Fig. 3:**
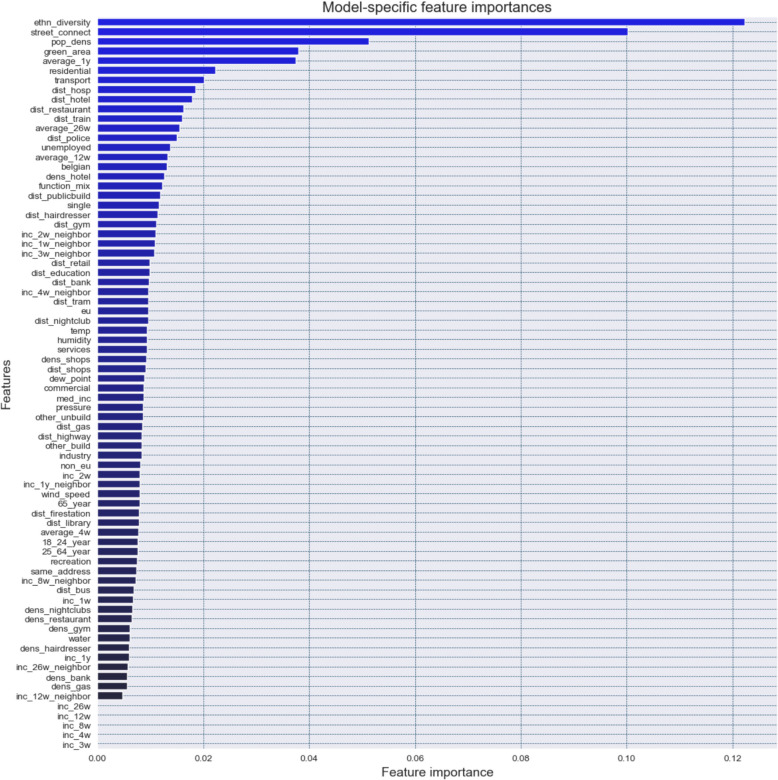
Model-specific feature importances derived from applying the XGBoost algorithm to the training data

The global model-specific feature importances derived from applying the XGBoost algorithm to the training data are shown in Fig. 3 (i.e., impurity or gain-based importances). In general, ethnic diversity and street connectivity significantly exhibit higher importance compared to the other features included in the model. In addition, population density as well as the percentage of green and residential area, transport infrastructure and the average one-year trend in residential burglary are also of higher importance. This implies that overall, these features are more likely to be used when the data is split in decision nodes throughout training process, resulting in increased information gain. Features with higher gain scores result in a greater reduction in impurity (i.e., they create more homogeneous child nodes), meaning they provide the most useful information for the model to distinguish between high and low burglary risk areas.

#### Permutation-based feature importances

**Fig. 4:**
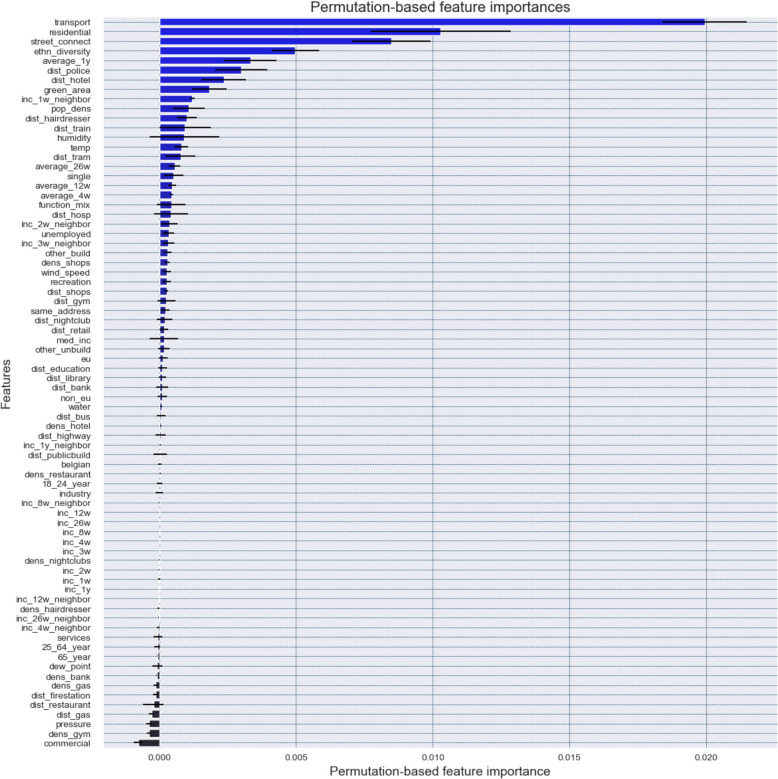
Permutation-based feature importances derived from applying the trained XGBoost model to the test dataset

Figure 4 presents the permutation-based feature importances. A higher value indicates a greater decrease in AUC on the test dataset, implying that a feature is highly relevant to the model’s performance, as randomly shuffling or permuting its values reduces the model’s ability to distinguish between high- and low-risk areas. In general, the most important features are the percentage of transport infrastructure, residential area, and street connectivity, followed by ethnic diversity, the one-year residential burglary trend, and the distance to the nearest police station. This differs somewhat from the model-specific importances, where ethnic diversity and street connectivity ranked highest. The prominence of transport infrastructure suggests it has a greater impact on unseen data than initially indicated. However, the overall importance scores are relatively low, likely due to feature redundancy. Permutation-based importance can be biased by correlated features, as shuffling one feature while keeping others fixed can create unrealistic data points, distorting importance estimates. Also, when two highly correlated features contribute similarly to the model’s predictions, feature importances may also be artificially diluted, as the model distributes predictive value between the features. This could explain why some features show lower-than-expected importance despite their relevance to predicted burglary risks.

#### SHAP-based feature importances

**Fig. 5:**
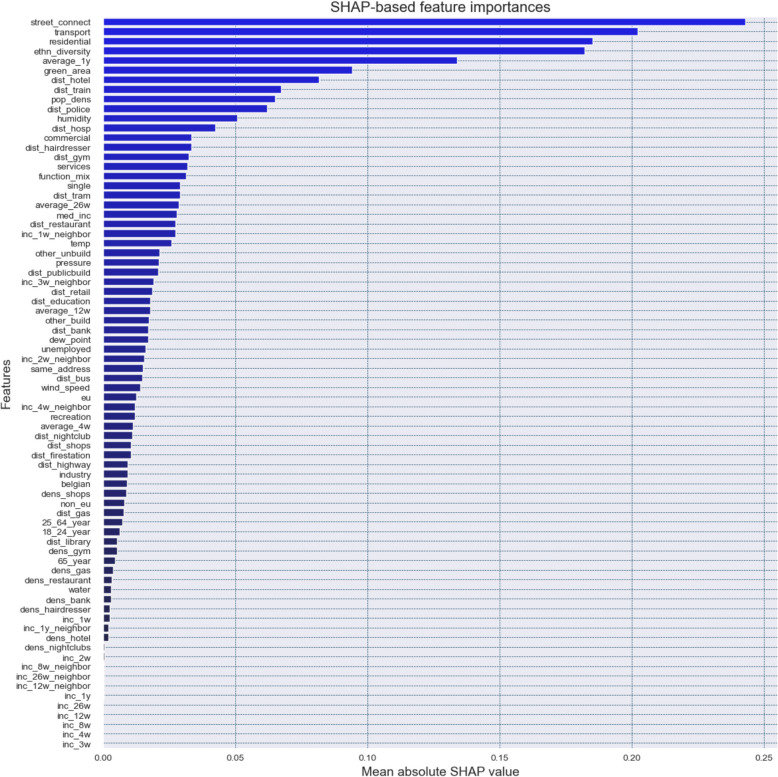
SHAP-based feature importances derived from applying the trained XGBoost model to the test dataset

In addition to the model-specific and permutation-based feature importances, Fig. 5 presents the feature importances derived from computing the mean absolute SHAP value for each feature. These SHAP values generally represent the average magnitude of each feature’s attribution to the prediction. Compared to the previous two feature importance measures, a slightly different picture emerges. Street connectivity now emerges as the highest contributing feature, followed by the percentage of transport infrastructure and residential area within a grid cell, as well as ethnic diversity, the average one-year trend in residential burglary, and the percentage of green area. Nevertheless, some overlap exists between the most important features identified across the different methods, particularly when comparing SHAP-based importances to the permutation-based feature importances. This relative consistency across methods also demonstrates the overall importances attached by the model to certain features, such as street connectivity, land usage (e.g., percentage of transport infrastructure and residential area) and ethnic diversity.

### Feature effects and interactions

#### Direction and magnitude of feature effects

**Fig. 6:**
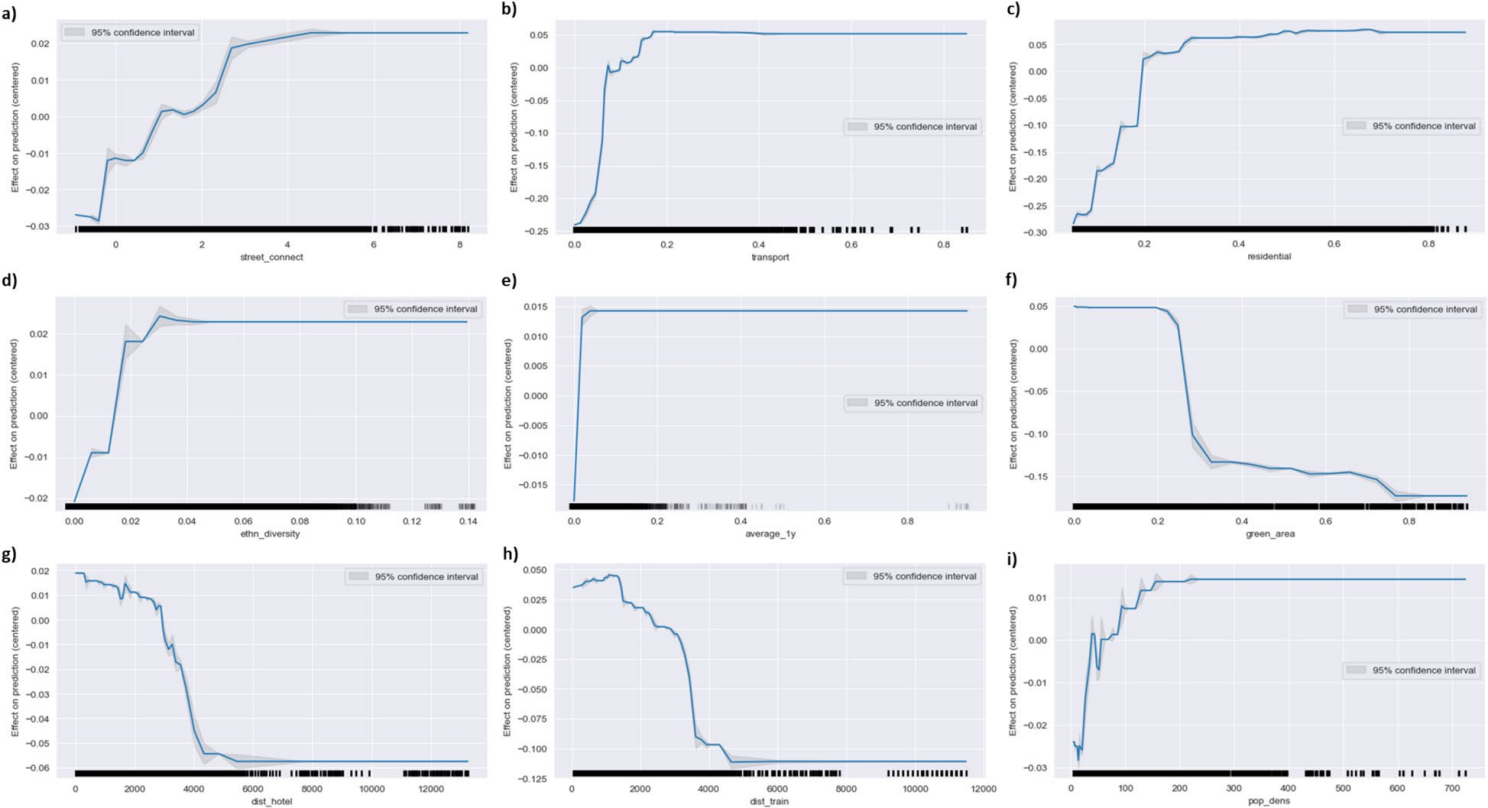
One-dimensional Accumulated Local Effect (ALE) plots for features with the highest global SHAP-based importance

**Fig. 7:**
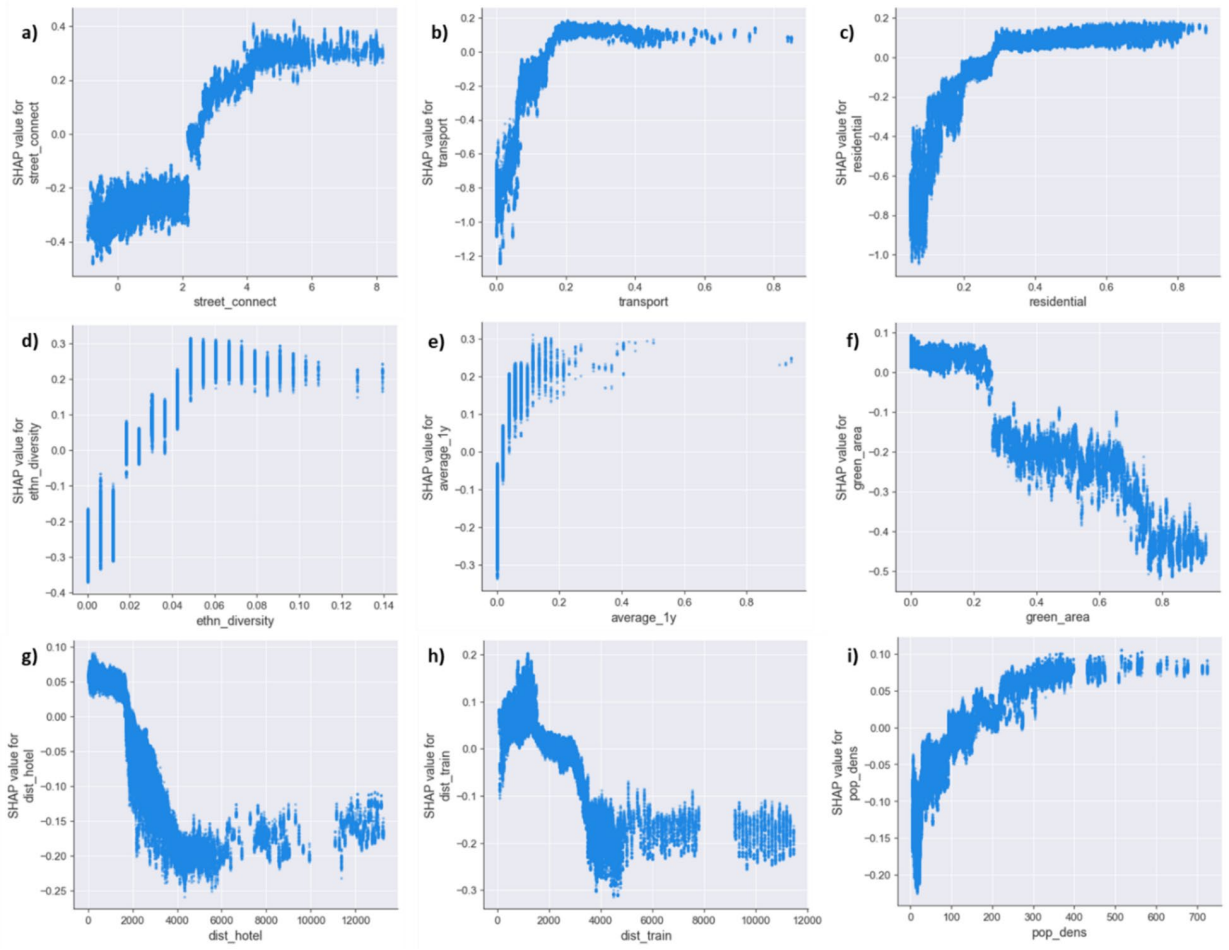
One-dimensional SHAP dependence plots for features with the highest global SHAP-based importance

In addition to assessing global feature importance, we examine how specific features influence the model’s predictions using one-dimensional ALE and SHAP dependence plots (Figs. 6 and 7). Both methods generally indicate that the most contributing features influence burglary risk in the same direction and exhibit non-linear relationships.

Specifically, the ALE plot shows that street connectivity is characterized by an increasing function in relation to predicted burglary risk (Fig. 6a). However, this effect appears to plateau at moderate street connectivity scores (beyond 4), suggesting that while enhanced street connectivity may initially elevate the predicted burglary risk, the marginal increase in risk diminishes beyond moderate street connectivity levels, indicating the presence of a threshold effect. Similar non-linear, positive relationships with diminishing returns are observed for transport infrastructure, residential area, ethnic diversity, and population density (Fig. 6b–d, i). These threshold effects may reflect the presence of outliers, where a long interval between two points in the feature space suggests that the ALE plot is interpolating more of these values and that estimates become less reliable. This is particularly visible in the average one-year burglary trend (Fig. 6e), which shows a steep increase at lower values but flattens out at higher values. Conversely, green area percentage and proximity to the nearest hotel and train station show negative, non-linear relationships with predicted burglary risk, with effects that also appear to plateau beyond specific values, indicating threshold effects in the opposite direction (Fig. 6f–h).

Compared to the ALE plots, the one-dimensional SHAP dependence plots (Fig. 7a – i) generally show the same non-linear relationships between the selected features and residential burglary risk. Hence, based on both the ALE and SHAP plots, the overall trends for these key features remain comparable: higher values on street connectivity, percentage residential area, percentage transport infrastructure, and ethnic diversity increase the predicted risk of residential burglary, while higher values on proximity to hotels, train stations, and the percentage of green decrease the predicted risk. In this regard, however, SHAP plots provide a more granular picture and allow capturing localized variations and outliers that are smoothed over in the ALE plots. This granularity is particularly relevant when examining for potential interactions, as SHAP plots allow to investigate the dispersion of individual instances along the SHAP values. As one-dimensions ALE plots average other feature’s effects, they might struggle to attribute individual effects to individual features, which could potentially bias the results, challenging the interpretation of individual effects.

#### Interactions between features

**Fig. 8:**
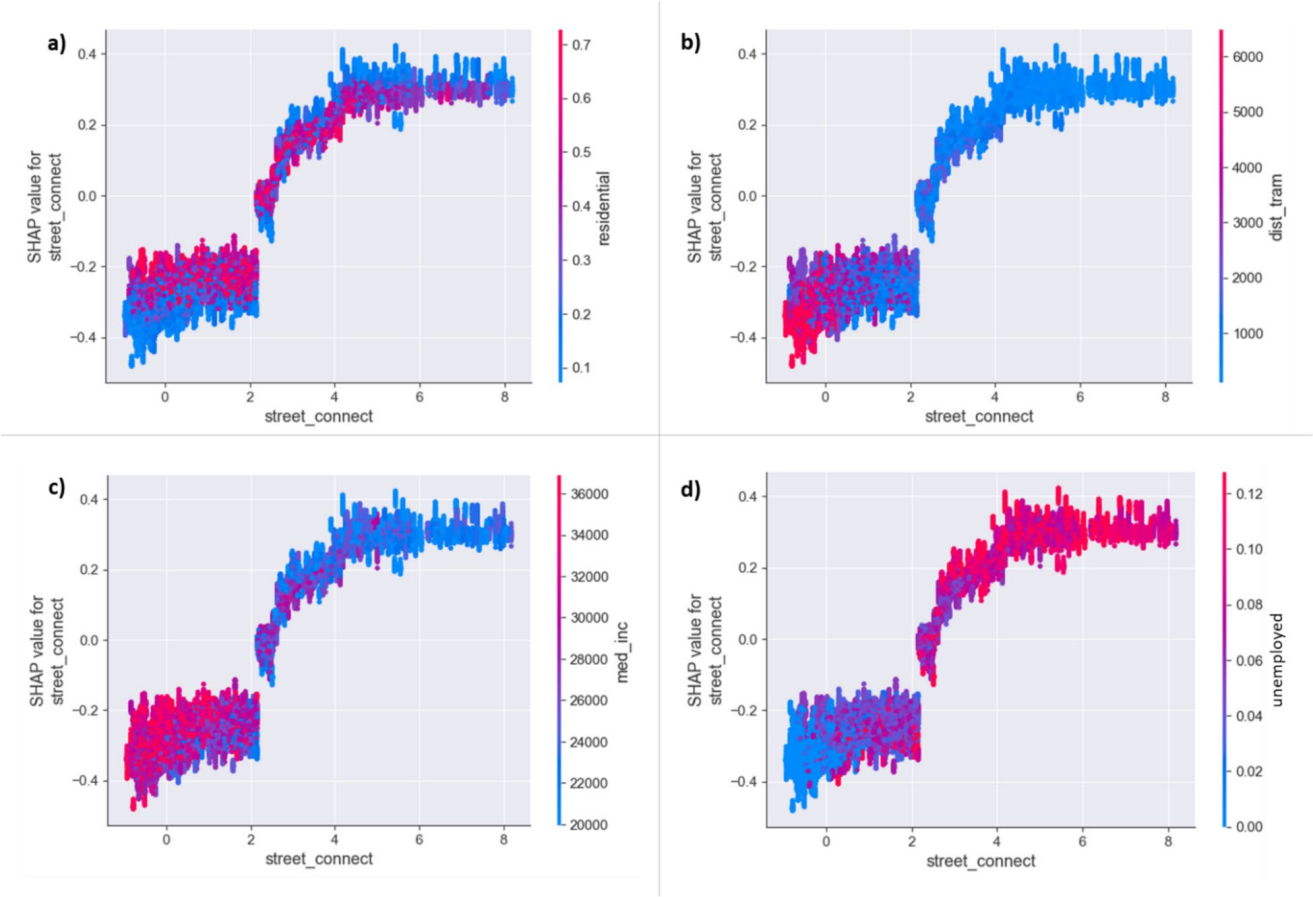
SHAP dependence plots for street connectivity colored by (**a**) percentage residential area in a grid cell, (**b**) distance to the nearest tram stop, (**c**) median income in a grid cell and (**d**) unemployment rate

**Fig. 9:**
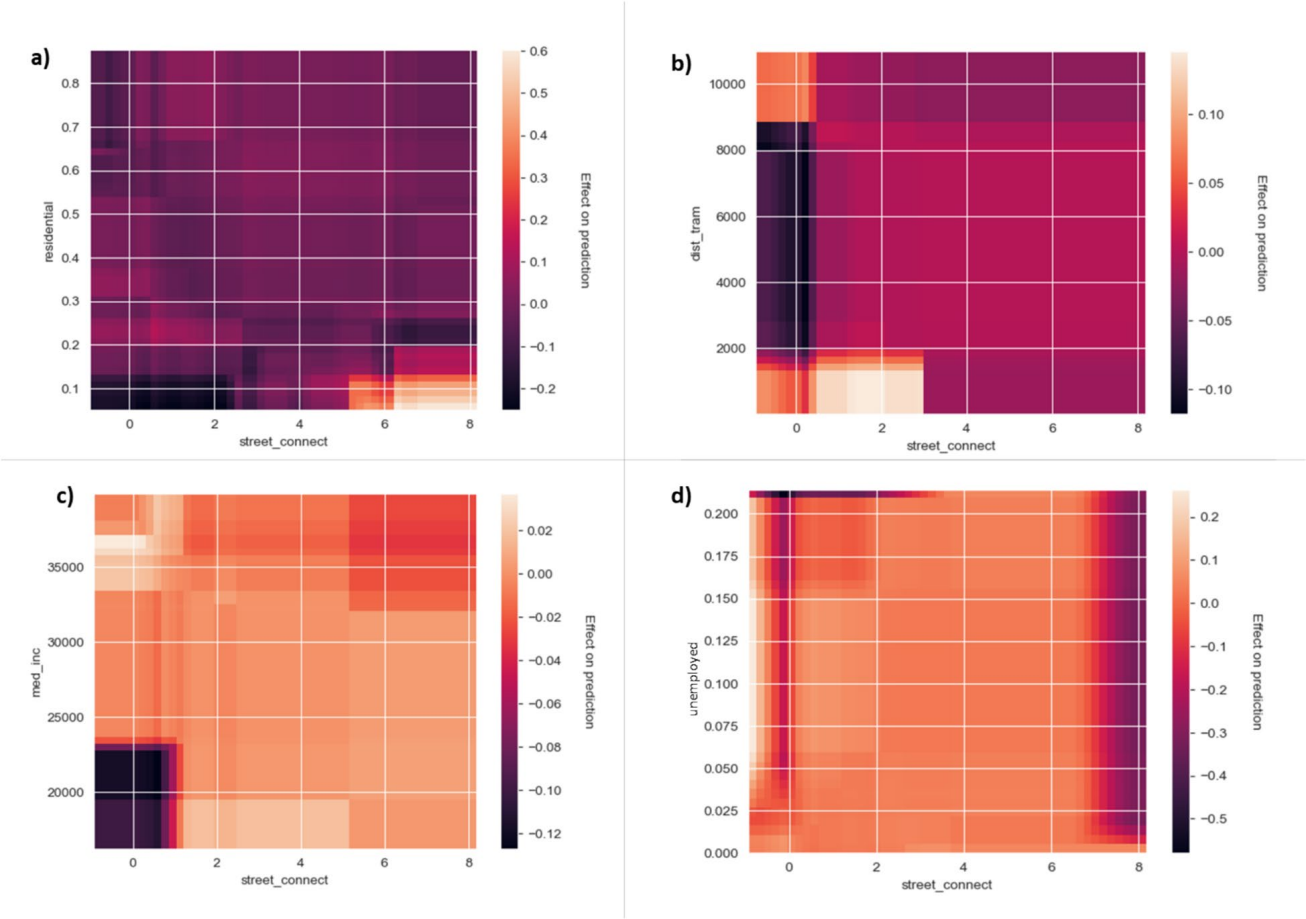
Two-dimensional ALE plots between street connectivity and (**a**) percentage residential area in a grid cell, (**b**) distance to the nearest tram stop, (**c**) median income in a grid cell and (**d**) unemployment rate

Figures 8 and 9 further show SHAP dependence plots and two-dimensional ALE plots, respectively, illustrating the interaction between street connectivity – one of the most important features – and four other key features that appear to have the largest interaction effect with street connectivity scores: percentage residential area in a grid cell, distance to the nearest tram stop, median income in a grid cell and unemployment rate.

The SHAP dependence plots reveal localized interaction effects between street connectivity and the selected features. In this regard, interaction effects between features can be best interpreted by looking at the vertical dispersion in the SHAP values for fixed variable values. As can be seen from Fig. 8, the interaction effects between street connectivity and the selected features are not uniformly strong or obvious across the entire feature space but appear more localized, affecting specific ranges of feature values. For example, in grid cells characterized by lower levels of street connectivity, a lower percentage of residential area is linked to lower predicted burglary risks (Fig. 8a), while in moderately to highly connected areas, a lower percentage of residential area increases the predicted burglary risk. Furthermore, in grid cells with lower street connectivity scores, greater distance to tram stops negatively impacts predicted burglary risk, likely because limited public transport access further restricts offender mobility (Fig. 8b). Conversely, in well-connected areas, closer proximity to tram stops increases burglary risk, which could indicate that the combination of high street connectivity and easy access to public transport may provide even more opportunities for (potential) offenders to navigate throughout the area. Interaction effects are also observed with socio-economic factors: higher median income decreases burglary risk in poorly connected areas, while lower median income increases risk in more connected areas (Fig. 8c). Similarly, lower unemployment rates reduce burglary risk in areas characterized by poor street connectivity, while higher unemployment rates increase it in highly connected areas (Fig. 8d).

The two-dimensional ALE plots (Fig. 9) provide a complementary but more averaged picture of feature interactions compared to the SHAP plots.^7^ The interaction between street connectivity and the percentage of residential area (Fig. 9a) aligns with the SHAP analysis, confirming that low street connectivity and low residential coverage reduce predicted burglary risk. Moreover, it can also be observed that at higher street connectivity levels, lower residential area coverage has a positive effect on predicted burglary risk. However, the interaction between street connectivity and distance to the nearest tram stop is less apparent in the ALE plot, with a positive effect only observed at connectivity scores between 1 and 2.5 (Fig. 9b), which is not reflected in the SHAP plot. Additionally, the ALE plots reveal a negative effect at low street connectivity combined with low median income (Fig. 9c), and suggest that at very low connectivity, higher unemployment significantly increases burglary risk beyond a certain threshold (Fig. 9d).

#### Local interpretability

**Fig. 10:**
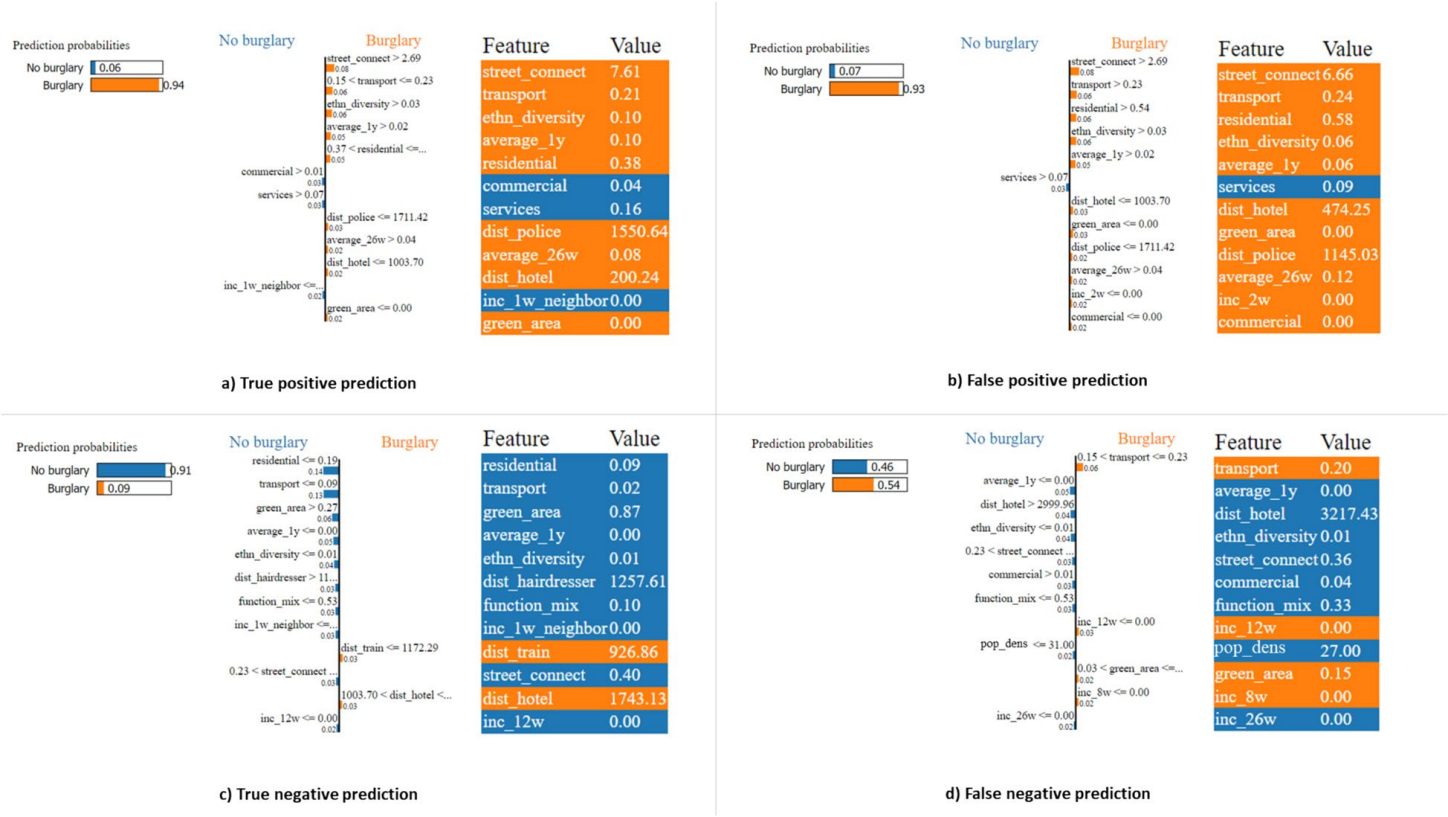
LIME output to explain true and false positive predictions as well as true and false negative predictions for one specific week throughout the 2018 test dataset

**Fig. 11:**
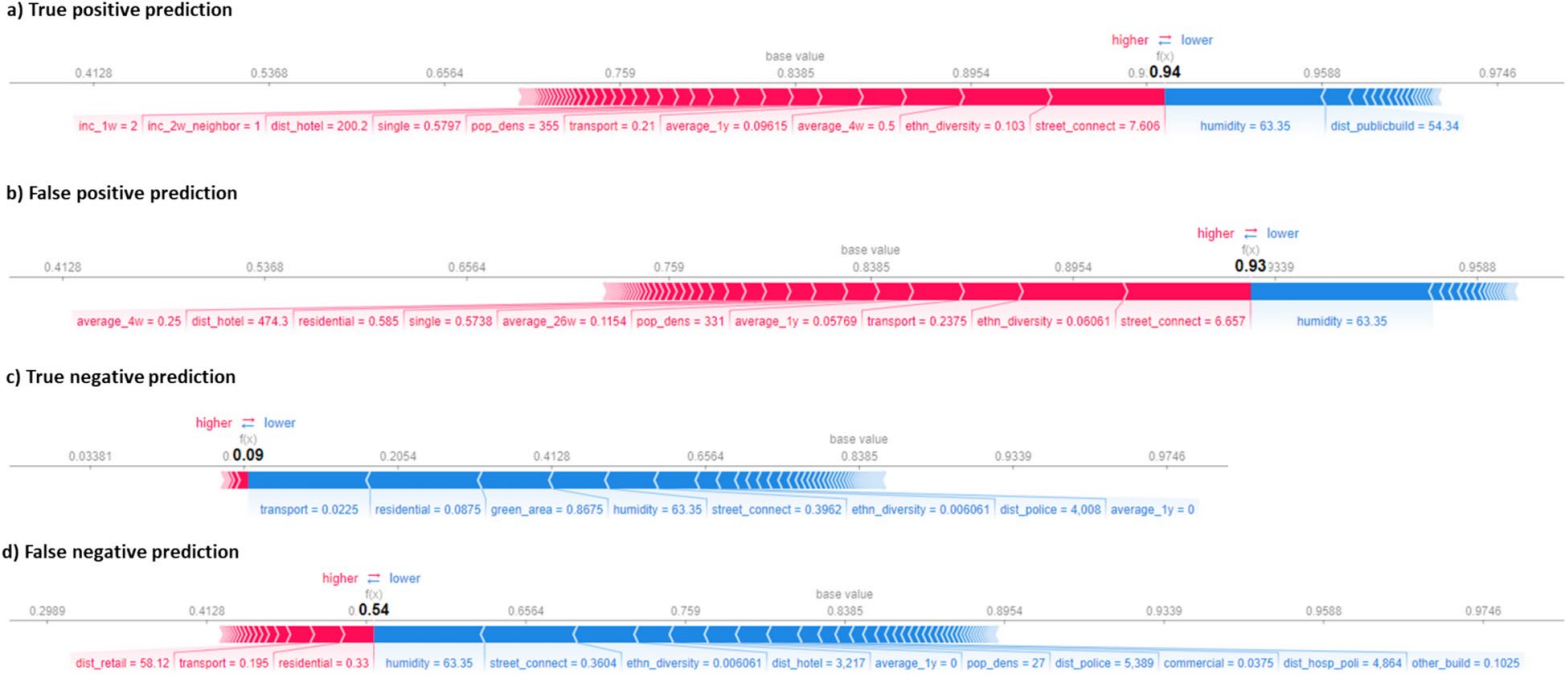
SHAP output to explain true and false positive predictions as well as true and false negative predictions for one specific week throughout the 2018 test dataset

Finally, Figs. 10 and 11 present the local feature importance scores derived using LIME (Fig. 10) and SHAP (Fig. 11) for four predicted instances: a true positive, false positive, true negative, and false negative prediction. These predicted instances, selected from a random week in 2018, are used to illustrate potential differences in local explanations provided by both methods. The instances are classified based on the predicted probability from the top 5% of grid cells with the highest predicted burglary risk for that week.^8^

For both the true and false positive predictions, both LIME and SHAP indicate that street connectivity and ethnic diversity push the predicted probability of residential burglary to a higher value. LIME also highlights the significant effect of transport infrastructure on the predicted probability, however, while SHAP attributes less importance to this feature. This suggests that the local impact of transport infrastructure might be more context-dependent and less globally influential across all predicted instances. SHAP additionally shows that both short- and long-term historical burglary trends contribute to higher predicted burglary risks, a pattern not observed in LIME, which only considers long-term burglary trends influencing the predicted probabilities. In addition, LIME does not attribute a negative effect of humidity on predicted burglary risk, whereas SHAP does.

In addition, for both the true and false negative predictions, features that increase the predicted probability for the true and false positive instances at a higher value also tend to decrease the predicted probability at lower values (Figs. 10c, d, 11c, d).^9^ For instance, lower values of street connectivity and ethnic diversity decrease the predicted probability. This links with the prior discussed threshold effects (Figs. 7, 8) which underline that the feature contribution is finite and beyond a specific value threshold do not seem to further affect the model’s predictions. This suggests that the model reacts to changes in feature values but only up to a certain point. However, SHAP and LIME also indicate differences in feature contributions. Apart from transport infrastructure, the features contributing positively to the predicted probability of a burglary in the false negative instance differ (Figs. 10d, 11d). While the type of features and the extent of their contribution in SHAP share similarities to LIME, specific features seem to contribute in the SHAP output which are not exhibiting high feature importance for LIME. For example, for all SHAP outputs, humidity pushes the probability lower, and even has the highest negative contribution for the true and false positive and the false negative; however, not for the true negative. A grid cell with lower values for features could therefore enhance the risk for a false negative. Specifically, lower values for specific features seem to influence the probability of a burglary negatively (such as less transport infrastructure and residential land use), together with higher values for green areas.

## Discussion of the results

This study contributes to the evolving body of research on interpreting machine learning-based crime predictions by empirically comparing the application of different methods that pertain to the field of XAI. In doing so, this study examined the interpretability of weekly micro-geographic residential burglary predictions derived through machine learning within a European setting (Ghent, Belgium), focusing on three specific levels of model interpretability, namely 1) global feature importances, 2) global feature effects (encompassing direction and magnitude) and interactions, and 3) local interpretability. In this regard, several key findings can be highlighted.

### Global feature importances

The first level of our analysis demonstrates that only a limited set of features, primarily related to the built environment and land usage, emerge as most important contributors to predicting weekly residential burglary hot spots in Ghent. This finding aligns with previous studies that have examined feature importances within the context of crime prediction (e.g., Kim & Lee, 2023; Wheeler & Steenbeek, 2021; Xie et al., 2022; Zhang et al., 2022, 2023). Importantly, our finding is reinforced through different methodological approaches. Especially when applied to unseen test data, permutation-based feature importance testing shows decreased model performance when these features are randomly permuted, while SHAP-based importance scores indicate consistent changes in conditional expectations for these features. In contrast, model-specific (gain-based) feature importances should be interpreted with caution as they cannot be validated on independent hold-out data in the same way as permutation- and SHAP-based feature importances.

However, the relative importance of built environment features across all tests suggests that they play an important role in determining predicted burglary risks, supporting established criminological theories and prior empirical work (e.g., Davies & Johnson, 2015; He et al., 2017; Hipp et al., 2021; Kim & Hipp, 2021; Zhang et al., 2022). Routine activity theory (Cohen & Felson, 1979) posits that crime occurs when motivated offenders, suitable targets, and the absence of capable guardians converge in space and time, while Crime Pattern Theory (Brantingham & Brantingham, 1993a, 1993b) furthermore suggests that people, including offenders, develop awareness spaces through their routine activities and thus become aware of potential crime opportunities through their routine activities. These (situational) convergent processes have found to be significantly influenced by features of the built environment, also in relation to residential burglary. These features include the configuration of the street network (e.g., Davies & Johnson, 2015), the presence of specific crime generators such as public transportation hubs (e.g., Bernasco & Block, 2011; Yue & Zhu, 2021) and hotels (e.g., Jones & Pridemore, 2019), crime attractors and target density such as residences in case of residential burglary (Vandeviver & Bernasco, 2020), crime detractors such as police stations (e.g., Blesse & Diegmann, 2022) and other elements of spatial lay-out such as green space (e.g., He et al., 2017). These findings also help to explain the spatial patterns observed in the predicted hot spots presented in Appendix B. Specifically, the consistent prominence of built environment features, such as street connectivity, land use intensity, and proximity to transport infrastructure, corresponds closely with characteristics typically concentrated in the city center, where most of the burglary hot spots are predicted.

Further, when examining the influence of features related to social disorganization theory, only ethnic diversity emerged as primary contributor to the model’s predictions. Given the limited body of research examining the influence of social cohesion and social disorganization on crime at micro-geographic scales (Jones & Pridemore, 2019), these findings can be contextualized using neighborhood-level insights. Drawing from social disorganization literature, it can be assumed that higher levels of ethnic diversity or heterogeneity impede social interactions and informal social control, resulting in reduced levels of guardianship and decreased chance of detection, conditions which are often preferred by burglars (Bernasco & Nieuwbeerta, 2005; Sampson & Grove, 1989; Vandeviver & Bernasco, 2020). These findings may also align with the spatial distribution of the predicted hot spots (Appendix B), as areas with higher predicted burglary risks often overlap with inner-city areas that are not only structurally dense but also ethnically diverse. In contrast, other features traditionally linked to social disorganization theory, such as residential instability, and indicators of concentrated disadvantage, such as income inequality, the percentage of single households, and unemployment rates, contributed less to the predicted burglary risks. Similarly, among time-varying features, only average long-term burglary trends seem to demonstrate substantial contribution to predictions, suggesting that burglars are likely to return to familiar areas where they have successfully offended before.

### Global feature effects and interactions

At the second level, both ALE and SHAP demonstrate strong convergent validity in mapping feature effects and interactions. Non-linear increasing effects were consistently observed for key features such as street connectivity, transport infrastructure, residential area, ethnic diversity, one-year burglary trends, and population density, all positively contributing to predicted burglary risk. These findings align with prior work, suggesting for example that greater street connectivity and transport infrastructure may enhance offender mobility and access to potential targets (e.g., Davies & Johnson, 2015; Frith et al., 2017, while increased target density (e.g., higher percentage residential area, higher population density) has been associated with a higher likelihood of burglary, as burglars tend to target areas with more potential targets (Vandeviver & Bernasco, 2020). In addition, ethnic diversity has also been linked to weaker social cohesion and reduced informal social control, factors that are associated with increased burglary risk (Sampson & Grove, 1989; Vandeviver & Bernasco, 2020). Also, the effect of the one-year burglary trend suggests that while short-term repeat victimization risks may be high immediately after a burglary, long-term trends may also play an important role in predicting future burglaries, potentially due to risk-heterogeneity where persistent, underlying risk factors contribute to ongoing burglary risks, with prior burglaries serving as an indicator of these stable vulnerabilities (e.g., Johnson, 2008).

In contrast, proximity to tram stops, train stations, and hotels, as well as the percentage of green area in grid cells, exhibited declining non-linear relationships. This suggests that burglary risk may increase as the distance from transportation hubs decreases, potentially due to increased area and target accessibility. Transportation nodes may also act as activity nodes that shape offenders' awareness spaces and facilitate access to potential targets, while providing opportunities for escaping (Yue & Zhu, 2021). Hotels may increase burglary risk as a crime generator, facilitating the convergence of motivated offenders, suitable targets, and the absence of capable guardians in space and time; particularly if the (nearby) presence of hotels implies more transient activity, fewer local residents, and lower informal social control, thereby reducing territoriality and increasing offender anonymity, as proposed by the so-called ‘enclosure hypothesis’ (Newman, 1972; Yue & Zhu, 2021). The presence of green space also appears to act as a deterrent for residential burglary, although the relationship between green space and crime remains underexplored and may depend on contextual factors such as design, usage, and maintenance (Wang et al., 2024).

The observed effects of the selected features do not show steady increases, however, but rather plateau at certain points, especially for higher predicted burglary risks. Beyond these thresholds, further changes in feature values no longer significantly impact predicted burglary risk. For instance, street connectivity demonstrates a non-linear relationship with predicted burglary risk, aligning with prior insights regarding the effect of street network configurations on (residential) burglary risks (e.g., Birks & Davies, 2017; Davies & Johnson, 2015). Given that burglary targets are fixed elements of the built environment, street network characteristics influence both offender and guardian movement through area permeability, affecting the accessibility of potential targets (Johnson & Bowers, 2010). According to crime pattern theory (Brantingham & Brantingham, 1993a, 1993b), the street network also shapes the ‘environmental backcloth’ that influences where and when crimes occur by guiding offenders’ routine activities and the development of awareness spaces. This could explain the observed positive relationship between higher street connectivity and increased burglary risk, as offenders are likely more familiar with these well-traveled routes and potential targets. Additionally, highly connected areas may also provide increased opportunities for escaping the area, thus reducing the perceived risk of apprehension (Davies & Johnson, 2015). Notably, the majority of burglaries are observed in Ghent's city center, characterized by its medieval core with high street connectivity. This historical layout, with narrow, interconnected streets, may facilitate offender movement and target accessibility, contributing to the heightened predicted burglary risks in these areas. However, increased street connectivity may also suggests more human activity and informal guardianship due to heightened visibility and ‘eyes on the street’ (Jacobs, 1961), which could counteract the effects of increased offender awareness. Our findings suggest that the positive effect of street connectivity plateaus at certain values, potentially due to such a counteracting effect. However, the willingness and ability of passers-by to intervene represents a complex issue and may be potentially limited, especially in transient areas (Reynald, 2010), where increased movement may also weaken territoriality, thereby increasing target vulnerability (Davies & Johnson, 2015).

This complexity is further demonstrated through the observed interactions between street connectivity and the percentage residential area in a grid cell, for example. Specifically, at higher levels of street connectivity, a lower percentage of residential area is associated with increased predicted burglary risk. This finding somehow aligns with prior empirical findings (e.g., Davies & Johnson, 2015; Frith et al., 2017; Newman, 1972) as well as with the spatial patterns of the predictions observed in Appendix B, as both high street connectivity and lower percentage of residential area characterizes the city center of Ghent. Higher street connectivity may indicate greater street usage for routine activities, expanding offenders' awareness spaces and familiarity with potential targets. Simultaneously, a lower percentage of residential units suggests fewer local guardians who could otherwise monitor their surroundings and intervene. This combination may create conditions where motivated offenders encounter suitable targets with minimal risk of detection. These findings support the idea that burglars prioritize locations that minimize the risk of apprehension. Empirical studies indicate that burglars prefer areas with more non-local passersby, where their presence is less conspicuous (e.g., Bennett & Wright, 1984). This challenges Jacobs’ (1961) eyes on the street'hypothesis, as it suggests that effective guardianship is not solely determined by pedestrian volume but also by the characteristics of those present (Davies & Johnson, 2015; Newman, 1972). Transient activity may therefore also weaken territoriality (enclosure hypothesis), making locations with high connectivity but low residential presence particularly attractive for burglars. This interpretation is further supported by observed interactions between street connectivity and social disorganization indicators, such as median income and unemployment rates. The increased burglary risk observed in lower-income and high-unemployment areas with high street connectivity suggests that socio-economic disadvantages may weaken informal social control, reduce guardianship effectiveness (Birks & Davies, 2017; Davies & Johnson, 2015; Sampson & Raudenbush, 1999), or reflect poor place management (Douglas & Welsh, 2020).

### Local interpretability

At the third level, this study examined four local scenarios (true and false positive and negative predictions) to assess how different XAI methods provide instance-level interpretability and compare in this regard. This analysis aimed to identify not only the features contributing to correct predictions but also those influencing assumed errors. While SHAP and LIME showed some similarities in identifying key features, they also produced differing interpretations. For example, SHAP highlighted the average number of burglaries in the past four weeks as a key contributor to a true positive prediction, whereas LIME only highlighted long-term burglary trends as key contributor. These observed differences can be potentially linked to the methodological differences between LIME and SHAP.

In general, LIME provides localized interpretability by approximating the model’s behavior with a simpler (linear) surrogate model, such as a linear regression model, around each predicted instance (Molnar, 2020). It derives these local approximations by perturbing the input data and assuming that the model behaves linearly within that small region. However, this reliance on independent perturbations ignores potential feature dependencies and interactions, which can lead to biased importance scores, particularly in models where feature relationships are non-linear, as observed in this study. Additionally, LIME's weighting scheme, which assigns importance to perturbed samples based on their proximity to the original instance, introduces another layer of variability (Alvarez-Melis & Jaakkola, 2018). Moreover, LIME does not have a completeness constraint, possibly impacting specific explanations within the context of sparsity. It could consequently perturb the data in a way that does not correspond to the actual data distribution, undermining the robustness of the explanations and increasing the risk of misleading explanation that could further misinform users, both in terms of resource allocation and exercising oversight (Ribeiro et al., 2016).

In contrast, SHAP offers a more theoretically grounded and robust framework for both local and global interpretability. By leveraging Shapley values from cooperative game theory, SHAP ensures a fair and consistent distribution of the model’s prediction across all features (Molnar, 2020). Unlike LIME, SHAP evaluates feature contributions across all possible feature subsets, allowing it to account for both individual effects and feature interactions. This makes SHAP better suited for models with complex relationships between predictors, which are clearly reflected in our findings. Furthermore, SHAP satisfies key desirable properties, such as local accuracy, consistency, and missingness, which strengthen the reliability of its explanations. Importantly, SHAP provides a unified framework where local explanations (instance-level) are consistent with global model understanding, enabling consistent interpretations across different levels (Molnar, 2020). This methodological properties likely contributed to the more stable and interpretable outputs observed in our findings, and underlines SHAP’s utility for applications where interpretability is essential to accountability, such as crime prediction.

### Limitations of the study

Several important limitations of this study warrant consideration. First, while this study is the first to apply XAI techniques to interpret short-term burglary predictions in a European context, however, its generalizability is limited to the urban setting of Ghent. While similar trends might be detectable in other middle-sized urban settings, the results should not be directly generalized to other geographic contexts without further validation. Second, by focusing exclusively on residential burglary, the broader applicability of our findings to other crime types remains uncertain, as different crimes may exhibit distinct spatiotemporal patterns and relationships with specific features. Crime-type specific explorations are consequently necessary. Third, this study employed only one machine learning algorithm (XGBoost) without comparing its performance to alternative prediction methods, such as other machine learning approaches (e.g., random forest, neural networks) or other methods that are more specifically designed for spatiotemporal data, such as spatiotemporal Cokriging (e.g., Yu et al., 2020). While XGBoost is a widely used algorithm for predictive modeling, different algorithms process feature interactions and non-linearities in distinct ways, which may lead to variations in both feature importance and prediction performance. Fourth, this study primarily relied on traditional data sources with limited temporal granularity, raising the question of whether integrating more fine-grained spatiotemporal data, such as mobility data, social media activity, or dynamic land use data, could enhance both predictive performance and model interpretability. Relatedly, the data only pertained to a specific period in time, so the results may reflect temporal patterns or situational conditions unique to that timeframe. As crime dynamics often fluctuate across seasons, years, or broader social and economic cycles, the generalizability of the findings beyond the study period remains uncertain. Fifth, the chosen spatial resolution of 200 by 200-m grid cells, while practical for predictive modeling, may have influenced the findings. Alternative spatial units of analysis, such as street segments or larger grid cells, could reveal different spatiotemporal dynamics, particularly concerning the role of street network configurations in determining the risk of crime. Finally, while XAI techniques provide valuable insights into how the model processes various features to generate predictions, these findings should not be mistaken for evidence of causal relationships between specific features and residential burglary occurrence. The model’s outputs may reflect patterns driven by correlations rather than underlying causal mechanisms, and the risk of overfitting specific (contextual) factors remains.

### Implications and avenues for future research and policy

Considering the findings and limitations of the present study, several implications and avenues emerge for future research and policy.

First, given that global feature importance rankings, as well as global feature effects and interactions, were largely similar across the different techniques evaluated in comparison with SHAP, especially among the top-contributing features, less computationally demanding approaches such as permutation-based feature importances (for feature importance) or ALEs (for feature effects and interactions) may offer a pragmatic substitute for SHAP in identifying global patterns in the model’s output, particularly when applied in large-scale real-time applications where computational efficiency is often warranted. The selection of an appropriate interpretable (XAI) method may therefore have more far-reaching implications at the local level than at the global level of model interpretability. Specifically, the findings show that substantial differences may emerge between SHAP and LIME in terms of capturing the influence of individual features on predicted burglary risks for specific places and periods in time. If different techniques highlight different local factors influencing crime risk at specific times and locations, the recommendations for targeted interventions may vary considerably. For instance, if SHAP identifies short crime patterns as a key local factor increasing predicted burglary risk, policymakers might prioritize interventions that specifically address repeat and near-repeat victimization, such as increasing police presence or implementing victim-support strategies. Conversely, if LIME fails to capture the relevance of short-term crime patterns, these short-term spatial contagion processes may be overlooked, potentially resulting in overemphasizing long-term interventions. Also, our observation that features with limited global influence may still have substantial local effects on predicted probabilities highlights the risk of relying solely on global feature importance rankings for feature selection processes. Future work should therefore also avoid excluding seemingly non-relevant features based on their low global importance, as this could lead to overlooking risks at specific locations and times, potentially impacting model performance and decision-making processes.

Second, the non-linear threshold effects and feature interactions found in this study suggest that future crime prevention strategies should account for the fact that burglary risk does not follow a simple linear relationship with specific features. Rather, specific features seem to interact with each other in ways that may only manifest under specific conditions, meaning interventions should be tailored accordingly. For example, in areas with high street connectivity, burglary risks may only rise when street connectivity exceeds a certain threshold and when it interacts with other features, such as low residential (population) density or high socioeconomic disadvantage. In such cases, crime prevention efforts could for example focus on enhancing natural surveillance, increasing local guardianship or strategically placing physical barriers to make it more difficult for burglars to access properties unnoticed. However, while many of the same factors contribute to elevated burglary risk in specific areas, it is also crucial to consider that burglaries can still occur in areas where these risk factors are less pronounced. This is clearly demonstrated by the false negative predicted instance as well as the spatiotemporal distribution of the burglary predictions (see Appendix B). The model consistently identifies burglary hot spots in the city center, but often fails to predict burglary risks in peripheral areas, particularly in the southwest of the city. This may indicate that the model captures the most dominant discriminative patterns but underperforms in areas where crime is less concentrated, yet still present. Such imbalance reflects a known limitation of predictive models: they tend to generalize well for patterns that occur most frequently, which can lead to the underrepresentation of more sporadic but equally relevant crime occurrences. To address this, spatially differentiated modelling may be necessary, such as training separate models for inside and outside city center areas. Such an approach has shown to improve crime prediction performance (see e.g., Rummens & Hardyns, 2020) and could thus also help ensure that crime prevention efforts based on crime predictions are equitably distributed across the urban landscape. Future work should therefore explore whether and to what extent the choice of XAI method affects the interpretation of predictions when applied to spatially differentiated machine learning models for different sub-areas within a setting.

Third, future work should examine to what extent the findings of this study generalize beyond the specific data, methods, and geographic context in which they were derived. The observed influence of street connectivity, for example, may differ across geographic settings and contexts due to variations in urban morphology. European cities such as Ghent typically exhibit irregular street patterns with unequal street lengths, whereas many non-European cities are characterized by gridiron layouts with more uniform street networks, which might influence offender movement, the development of awareness spaces, and target selection differently. Additionally, structural differences in socio-demographic- and -economic structures, land-use patterns, and crime prevention strategies may further shape burglary dynamics in ways that affect the applicability of these findings elsewhere. For example, although ethnic heterogeneity has often been linked with higher crime rates, commonly attributed to reduced social cohesion and informan social control, this relationship might be context-dependent given differing immigration histories and integration policies. The inclusion of such sensitive features, or related proxies, in predictive models also raises important ethical considerations, as they risk reinforcing social biases if not carefully contextualized. Future research should therefore assess both the theoretical and empirical relevance as well as the ethical implications of specific features and examine how their effects and interpretations may vary across diverse geographic settings and contexts. Likewise, future work should explore to what extent crime risk predictions and interpretations are consistent across different crime types, as the factors influencing burglary may differ substantially from those influencing the risk of other offenses such as robbery or assault, particularly when comparing urban, suburban, and rural settings. Relatedly, future work should examine to what extent different crime prediction methods, both machine learning algorithms and other methods specifically tailored to modeling spatiotemporal data (e.g. spatiotemporal Cokriging), result in differing crime risk interpretations and whether the same results are consistently obtained across prediction methods. A comparison should therefore be made along the continuum of the flexibility and interpretability of specific methods, ranging from less flexible but more transparent methods to more flexible yet less interpretable methods. This comparison could help to identify the trade-offs between model complexity and interpretability, guiding the selection of appropriate methods for specific research contexts. Also, future work should thereby examine whether the identified feature effects, interactions, and local interpretations remain stable when applied to data that pertain to different timeframes, including longer observation periods and data spanning multiple temporal contexts, to assess the temporal robustness of both predictions and explanations.

Finally, from a theoretical perspective, the findings highlight the importance of moving beyond examining the isolated effects of environmental and social area characteristics toward a more integrated understanding of their interactions and threshold effects. Future research should focus in particular on how configurations of the built environment interact with social processes in shaping criminal opportunity structures. This includes examining the conditional effectiveness of guardianship under varying environmental conditions, the influence of street configurations and land use on awareness spaces, and the role of social cohesion, collective efficacy, and social disorganization in the relationship between crime opportunity and crime risk. Advancing such integrated perspectives would not only strengthen theoretical dialogue between crime opportunity and social disorganization theories, but also inform the development of more context-sensitive and location-specific crime prevention strategies.

## Conclusion

This study compares different XAI techniques in interpreting short-term crime predictions, emphasizing their implications for both future research and policy. Among the methods evaluated, SHAP stands out for its ability to provide both global and local explanations of model behavior. However, its computational demands may necessitate a balance between complexity and practicality in real-world applications.​ By integrating technological advancements with criminological theory, this study demonstrates that machine learning interpretability can validate and extend traditional theoretical frameworks in criminology. Notably, the study reveals that different XAI techniques can yield varying explanations, which may influence the formulation of crime prevention recommendations. Therefore, it is essential to link the obtained explanations to the specific techniques used to ensure accurate and effective policy decisions.​ Future research should focus on developing more sophisticated yet efficient interpretability techniques that maintain transparency and equity. Such advancements will bridge the gap between predictive analytics and actionable crime prevention strategies, ensuring that predictive models contribute meaningful insights for evidence-based crime prevention.

## Supplementary Information

Supplementary Material 1.

## Data Availability

Not applicable.

## Footnotes

1 The geocoding process was primarily done using the official address reference database of the Flemish government. In cases where addresses could not be geocoded using this primary method, the ArcGIS geocoder API in Python was used. For crime incidents missing house numbers, incidents were geocoded to the middle of the corresponding street. Crime events without sufficient street information were eventually excluded from the analysis.

2 Both street connectivity and function mix scores were obtained from the walkability tool, see: https://www.gezondleven.be/settings/gezonde-gemeente/gezonde-publieke-ruimte/walkability-tool

3 Areal interpolation was done using the Tobler package in Python, version 3.11.: https://github.com/pysal/tobler

4 Hence, the target variable in this study involves a dichotomized variable: no burglary or burglary.

5 All analyses were conducted using Python, version 3.11.

6 The tuned hyperparameters are 1) the number of estimators, 2) the maximum depth of the decision trees, 3) the minimum child weight, 4) the learning rate, 5) the gamma parameter, 6) the subsample parameter, and 7) the ‘column sample by tree’ parameter.

7 Specifically, the two-dimensional ALE plots presented here consider the second-order effect of two features without including the main effects of the features.

8 From both Fig. 10 and Fig. 11, it is evident that the predicted probabilities are relatively high. This is likely because the model was trained on a randomly under-sampled training dataset, which may affect the a posteriori predicted probability by altering the a priori* probability* within the dataset, which can lead to biased probability estimates (e.g., Japkowicz & Stephen, 2002; Dal Pozzolo et al., 2015). For this study, we calibrated the predicted probabilities of the test set to ensure they more accurately reflected the actual probability of burglary (using Platt scaling calibration). However, when applying the different XAI techniques, it was not possible to use these calibrated probabilities. This limitation arose because all tests needed to be conducted using the base estimator, with calibration applied only after the training phase.

9 It is clear that the decision to have a cut-off contributed to the false negative here, i.e. only 94 grid cells with the highest probability are predicted at risk, which is why this grid cell was classified as ‘no burglary’. Nonetheless, the difference in the predicted probability needs to be addressed, as it is likely due to a different combination of types and values of features and the extent that the combination of these influence the prediction for these grid cells.

## References

[CR1] Agarwal, N., & Das, S. (2020). Interpretable machine learning tools: A survey. *IEEE Symposium Series on Computational Intelligence (SSCI)*, 1528–1534. 10.1109/SSCI47803.2020.9308260.

[CR2] Alvarez-Melis, D., & Jaakkola, T. S. (2018). On the robustness of interpretability methods. arXiv:1806.08049.

[CR3] Amarasinghe, K., Rodolfa, K. T., Lamba, H., & Ghani, R. (2023). Explainable machine learning for public policy: Use cases, gaps, and research directions. *Data & Policy,**5*, e5.

[CR4] Andresen, M. A., & Malleson, N. (2013). Crime seasonality and its variations across space. *Applied Geography,**43*, 25–35.

[CR5] Apley, D. W., & Zhu, J. (2020). Visualizing the effects of predictor variables in black box supervised learning models. *Journal of the Royal Statistical Society Series B: Statistical Methodology,**82*(4), 1059–1086.

[CR6] Bennett, T., & Wright, R. (1984). Burglars on Burglary: Prevention and the offender. *Gower,**14*, 73–113.

[CR7] Bernasco, W., & Block, R. (2011). Robberies in Chicago: A block-level analysis of the influence of crime generators, crime attractors, and offender anchor points. *Journal of Research in Crime and Delinquency,**48*(1), 33–57.

[CR8] Bernasco, W., Johnson, S. D., & Ruiter, S. (2015). Learning where to offend: Effects of past on future burglary locations. *Applied Geography,**60*, 120–129.

[CR9] Bernasco, W., & Nieuwbeerta, P. (2005). How do residential burglars select target areas? A new approach to the analysis of criminal location choice. *British Journal of Criminology,**45*(3), 296–315.

[CR10] Birks, D., & Davies, T. (2017). Street network structure and crime risk: An agent-based investigation of the encounter and enclosure hypotheses. *Criminology,**55*(4), 900–937.

[CR11] Blesse, S., & Diegmann, A. (2022). The place-based effects of police stations on crime: Evidence from station closures. *Journal of Public Economics,**207*, 104605.

[CR12] Bowers, K. J., & Johnson, S. D. (2004). Who commits near repeats? A test of the boost explanation. *Western Criminology Review,**5*, 12–24.

[CR13] Brantingham, P. L., & Brantingham, P. J. (1993a). Environment, routine and situation: Toward a pattern theory of crime. *Advances in Criminological Theory,**5*, 259–294.

[CR14] Brantingham, P. L., & Brantingham, P. J. (1993b). Nodes, paths and edges: Considerations on the complexity of crime and the physical environment. *Journal of Environmental Psychology,**13*(1), 3–28.

[CR15] Caplan, J. M., & Kennedy, L. W. (2016). *Risk terrain modeling: Crime prediction and risk reduction*. Univ of California Press.

[CR16] Chainey, S., Tompson, L., & Uhlig, S. (2008). The utility of hotspot mapping for predicting spatial patterns of crime. *Security Journal,**21*, 4–28.

[CR17] Chen, T., & Guestrin, C. (2016). Xgboost: A scalable tree boosting system. In *Proceedings of the 22nd acm sigkdd international conference on knowledge discovery and data mining* (pp. 785-794).

[CR18] Cohen, L. E., & Felson, M. (1979). Social change and crime rate trends: A routine activity approach. *American Sociological Review,**44*(4), 588–608.

[CR19] Corcoran, J., & Zahnow, R. (2022). Weather and crime: A systematic review of the empirical literature. *Crime Science,**11*(1), 16.

[CR20] Dal Pozzolo, A., Caelen, O., Johnson, R. A., & Bontempi, G. (2015). Calibrating probability with undersampling for unbalanced classification. In *2015 IEEE symposium series on computational intelligence *(pp. 159-166). IEEE.

[CR21] Datta, A., Sen, S., & Zick, Y. (2016). Algorithmic transparency via quantitative input influence: Theory and experiments with learning systems. In *2016 IEEE symposium on security and privacy (SP)* (pp. 598-617). IEEE.

[CR22] Davies, T., & Johnson, S. D. (2015). Examining the relationship between road structure and burglary risk via quantitative network analysis. *Journal of Quantitative Criminology,**31*, 481–507.

[CR23] Deng, Y., He, R., & Liu, Y. (2023). Crime risk prediction incorporating geographical spatiotemporal dependency into machine learning models. *Information Sciences,**646*, 119414.

[CR24] Douglas, S., & Welsh, B. C. (2020). Place managers for crime prevention: The theoretical and empirical status of a neglected situational crime prevention technique. *Crime Prevention and Community Safety,**22*, 99–109.

[CR25] Frith, M. J., Johnson, S. D., & Fry, H. M. (2017). Role of the street network in burglars’ spatial decision-making. *Criminology,**55*(2), 344–376.

[CR26] Haberman, C. P., & Ratcliffe, J. H. (2015). Testing for temporally differentiated relationships among potentially criminogenic places and census block street robbery counts. *Criminology,**53*(3), 457–483.

[CR27] Hardyns, W., & Khalfa, R. (2023). Predicting crime across cities and regions: A comparative analysis of predictive modelling in three Belgian settings. *Applied Spatial Analysis and Policy,**16*(1), 485–508.

[CR28] He, L., Páez, A., & Liu, D. (2017). Built environment and violent crime: An environmental audit approach using google street view. *Computers, Environment and Urban Systems,**66*, 83–95.

[CR29] Hipp, J. R., Lee, S., Ki, D., & Kim, J. H. (2021). Measuring the built environment with Google Street View and machine learning: Consequences for crime on street segments. *Journal of Quantitative Criminology,**38*, 1–29.

[CR30] Jacobs, J. (1961). *The life and death of Great American cities*. Random House.

[CR31] James, G., Witten, D., Hastie, T., spsampsps Tibshirani, R. (2013). *An introduction to statistical learning* (vol. 112, p. 18). Springer.

[CR32] Japkowicz, N., & Stephen, S. (2002). The class imbalance problem: A systematic study.* Intelligent data analysis, 6*(5), 429-449.

[CR33] Johnson, S. D., & Bowers, K. J. (2010). Permeability and burglary risk: Are cul-de-sacs safer? *Journal of Quantitative Criminology,**26*, 89–111.

[CR34] Johnson, S. D. (2008). Repeat burglary victimisation: A tale of two theories. *Journal of Experimental Criminology,**4*, 215–240.

[CR35] Johnson, S. D., Summers, L., & Pease, K. (2009). Ofender as forager? A direct test of the boost account of victimization. *Journal of Quantitative Criminology,**25*, 181–200. 10.1007/s10940-008-9060-8

[CR36] Jones, R. W., & Pridemore, W. A. (2019). Toward an integrated multilevel theory of crime at place: Routine activities, social disorganization, and the law of crime concentration. *Journal of Quantitative Criminology,**35*, 543–572.

[CR37] Kadar, C., Maculan, R., & Feuerriegel, S. (2019). Public decision support for low population density areas: An imbalance-aware hyper-ensemble for spatio-temporal crime prediction. *Decision Support Systems,**119*, 107–117.

[CR38] Kim, S., & Lee, S. (2023). Nonlinear relationships and interaction effects of an urban environment on crime incidence: Application of urban big data and an interpretable machine learning method. *Sustainable Cities and Society,**91*, 104419.

[CR39] Kim, Y. A., & Hipp, J. R. (2021). Density, diversity, and design: Three measures of the built environment and the spatial patterns of crime in street segments. *Journal of Criminal Justice,**77*, 101864.

[CR40] Kinney, J. B., Brantingham, P. L., Wuschke, K., Kirk, M. G., & Brantingham, P. J. (2008). Crime attractors, generators and detractors: Land use and urban crime opportunities. *Built Environment,**34*(1), 62–74.

[CR41] Kubrin, C. E., & Hipp, J. R. (2016). Do fringe banks create fringe neighborhoods? Examining the spatial relationship between fringe banking and neighborhood crime rates. *Justice Quarterly,**33*(5), 755–784.

[CR42] Laato, S., Tiainen, M., Najmul Islam, A. K. M., & Mäntymäki, M. (2022). How to explain AI systems to end users: A systematic literature review and research agenda. *Internet Research,**32*(7), 1–31.

[CR43] Mehta, M., Palade, V., & Chatterjee, I. (Eds.). (2023). *Explainable AI: Foundations, methodologies and applications*. Springer.

[CR44] Mohler, G. O., Short, M. B., Brantingham, P. J., Schoenberg, F. P., & Tita, G. E. (2011). Self-exciting point process modeling of crime. *Journal of the American Statistical Association,**106*(493), 100–108.

[CR45] Molnar, C. (2020). *Interpretable machine learning*. Lulu.com.

[CR46] Newman, O. (1972). *Defensible space*. Macmillan.

[CR47] Pease, K. (1998). *Repeat victimisation: Taking stock* (vol. 90). Home Office Police Research Group.

[CR48] Perkins, D. D., Wandersman, A., Rich, R. C., & Taylor, R. B. (1993). The physical environment of street crime: Defensible space, territoriality and incivilities. *Journal of Environmental Psychology,**13*(1), 29–49.

[CR49] Piza, E. L., Welsh, B. C., Farrington, D. P., & Thomas, A. L. (2019). CCTV surveillance for crime prevention: A 40-year systematic review with meta-analysis. *Criminology & Public Policy,**18*(1), 135–159.

[CR50] Reynald, D. M. (2010). Guardians on guardianship: Factors affecting the willingness to supervise, the ability to detect potential offenders, and the willingness to intervene. *Journal of Research in Crime and Delinquency,**47*(3), 358–390.

[CR51] Ribeiro, M. T., Singh, S., & Guestrin, C. (2016, August). " Why should i trust you?" Explaining the predictions of any classifier. In *Proceedings of the 22nd ACM SIGKDD international conference on knowledge discovery and data mining* (pp. 1135-1144).

[CR52] Rosser, G., Davies, T., Bowers, K. J., Johnson, S. D., & Cheng, T. (2017). Predictive crime mapping: Arbitrary grids or street networks? *Journal of Quantitative Criminology,**33*, 569–594. 32025086 10.1007/s10940-016-9321-x PMC6979510

[CR53] Rummens, A., Hardyns, W., & Pauwels, L. (2017). The use of predictive analysis in spatiotemporal crime forecasting: Building and testing a model in an urban context. *Applied geography, 86*, 255-261.

[CR54] Rummens, A., & Hardyns, W. (2020). Comparison of near-repeat, machine learning and risk terrain modeling for making spatiotemporal predictions of crime. *Applied Spatial Analysis and Policy,**13*(4), 1035–1053.

[CR55] Rummens, A., & Hardyns, W. (2021). The effect of spatiotemporal resolution on predictive policing model performance. *International Journal of Forecasting,**37*(1), 125–133.

[CR56] Rummens, A., Snaphaan, T., Van de Weghe, N., Van den Poel, D., Pauwels, L. J., & Hardyns, W. (2021). Do mobile phone data provide a better denominator in crime rates and improve spatiotemporal predictions of crime? *ISPRS International Journal of Geo-Information,**10*(6), 369.

[CR57] Sampson, R. J. (1985). Neighborhood and crime: The structural determinants of personal victimization. *Journal of Research in Crime and Delinquency,**22*(1), 7–40.

[CR58] Sampson, R. J., & Groves, W. B. (1989). Community structure and crime: Testing social-disorganization theory. *American Journal of Sociology,**94*(4), 774–802.

[CR59] Sampson, R. J., & Raudenbush, S. W. (1999). Systematic social observation of public spaces: A new look at disorder in urban neighborhoods. *American Journal of Sociology, 105*(3), 603-651.

[CR60] Sampson, R. J., Raudenbush, S. W., & Earls, F. (1997). Neighborhoods and violent crime: A multilevel study of collective efficacy. *Science,**277*(5328), 918–924. 9252316 10.1126/science.277.5328.918

[CR61] Shapley, L. S. (1953). 17. A value for n-person games. *Contributions to the Theory of Games (Am-28), II*, 307–318. 10.1515/9781400881970-018

[CR62] Shaw, C. R., & McKay, H. (1969). *Juvenile delinquency and urban areas*. University of Chicago Press.

[CR63] Snaphaan, T., & Hardyns, W. (2021). Environmental criminology in the big data era. *European Journal of Criminology,**18*(5), 713–734.

[CR64] Stiglic, G., Kocbek, P., Fijacko, N., Zitnik, M., Verbert, K., & Cilar, L. (2020). Interpretability of machine learning-based prediction models in healthcare. *Wiley Interdisciplinary Reviews: Data Mining and Knowledge Discovery,**10*(5), 1-13.

[CR65] Štrumbelj, E., & Kononenko, I. (2014). Explaining prediction models and individual predictions with feature contributions. *Knowledge and Information Systems,**41*, 647–665.

[CR66] Townsley, M., Homel, R., & Chaseling, J. (2003). Infectious burglaries: A test of the near repeat hypothesis. *British Journal of Criminology,**43*, 615–633. 10.1093/bjc/43.3.615

[CR67] Vandeviver, C., & Bernasco, W. (2020). “Location, location, location”: Effects of neighborhood and house attributes on burglars’ target selection. *Journal of Quantitative Criminology,**36*, 779–821.

[CR68] Vestby, A., & Vestby, J. (2021). Machine learning and the police: Asking the right questions. *Policing: A Journal of Policy and Practice,**15*(1), 44–58.

[CR69] Wang, R., Cleland, C. L., Weir, R., McManus, S., Martire, A., Grekousis, G., ... & Hunter, R. F. (2024). Rethinking the association between green space and crime using spatial quantile regression modelling: Do vegetation type, crime type, and crime rates matter?. *Urban Forestry & Urban Greening, 101*, 128523.

[CR70] Wang, Z., Liu, L., Haberman, C., Lan, M., Yang, B., & Zhou, H. (2021). Burglaries and entry controls in gated communities. *Urban Studies,**58*(14), 2920–2932.

[CR71] Weisburd, D. (2015). The law of crime concentration and the criminology of place. *Criminology,**53*(2), 133–157.

[CR72] Wheeler, A. P. (2019). Quantifying the local and spatial effects of alcohol outlets on crime. *Crime & Delinquency,**65*(6), 845–871.

[CR73] Wheeler, A. P., & Steenbeek, W. (2021). Mapping the risk terrain for crime using machine learning. *Journal of Quantitative Criminology,**37*, 445–480.

[CR74] Xie, H., Liu, L., & Yue, H. (2022). Modeling the effect of streetscape environment on crime using street view images and interpretable machine-learning technique. *International Journal of Environmental Research and Public Health,**19*(21), 13833. 36360717 10.3390/ijerph192113833 PMC9655263

[CR75] Yu, H., Liu, L., Yang, B., & Lan, M. (2020). Crime prediction with historical crime and movement data of potential offenders using a spatio-temporal cokriging method. *ISPRS International Journal of Geo-Information,**9*(12), 732.

[CR76] Yue, H., Liu, L., & Xiao, L. (2023). Investigating the effect of people on the street and streetscape physical environment on the location choice of street theft crime offenders using street view images and a discrete spatial choice model. *Applied Geography, 157,* 103025.

[CR77] Yue, H., & Zhu, X. (2021). The influence of urban built environment on residential burglary in China: Testing the encounter and enclosure hypotheses. *Criminology & Criminal Justice,**21*(4), 508–528.

[CR78] Zhang, X., Liu, L., Lan, M., Song, G., Xiao, L., & Chen, J. (2022). Interpretable machine learning models for crime prediction. *Computers, Environment and Urban Systems,**94*, 101789.

[CR79] Zhang, Y., Cai, L., Song, G., & Zhu, C. (2023). The Long-Term Theft Prediction in Beijing Using Machine Learning Algorithms: Comparison and Interpretation. *Crime & Delinquency, 0*(0), 1-31.

[CR80] Zheng, H., Yuan, J., & Chen, L. (2017). Short-term load forecasting using EMD-LSTM neural networks with a Xgboost algorithm for feature importance evaluation. *Energies,**10*(8), 1168.

