# Supplementary material for: Comparing XAI techniques for interpreting short-term burglary predictions at micro-places
Source: Comput Urban Sci. 2025 May 9;5(1):27. doi: 10.1007/s43762-025-00185-x (PMC12064634; doi:10.1007/s43762-025-00185-x)
Supplement: Supplementary file 1 — Supplementary Material 1. [file 43762_2025_185_MOESM1_ESM.docx]

# **Supplementary material**

# **Appendix A: Overview of features included in the machine learning model**

| **Data type** | **Name** | **Description** | **Source** |
| --- | --- | --- | --- |
| Crime data | inc_1w,  inc_2w,  inc_3w,  inc_4w,  inc_8w,  inc_12w,  inc_26w,  inc_1y | Number of incidents in the respective grid cells in the past period | Local police of Ghent (Belgium) |
|  | average_4w,  average_12w,  average_26w,  average_1y | Average number of incidents in the respective grid cells within a defined period |  |
|  | inc_1w_neighbor, inc_2w_neighbor, inc_3w_neighbor, inc_4w_neigbor, inc_8w_neighbor,  inc_12w_neighbor,  inc_26w_neighbor, inc_1y_neighbor | Number of incidents in the neighboring grid cells of each grid cell in the past period |  |
| Socio-demographic and -economic characteristics | pop_dens | Number of residents in a grid cell | Open data repository of the Flemish government |
|  | 18_24_year | Fraction of residents between 18-24 y/o |  |
|  | 25_64_year | Fraction of residents between 25-64 y/o |  |
|  | 65_year | Fraction of residents older than 65 y/o |  |
|  | same_address | Fraction of stable residential population |  |
|  | single | Faction of single households |  |
|  | non_eu | Fraction of residential population with a non-European origine |  |
|  | eu | Fraction of residential population with a European origine |  |
|  | belgian | Fraction of residential population with a Belgian origine |  |
|  | unemployed | Fraction of residential population that is unemployed |  |
|  | med_inc | Median income within a grid cell |  |
|  | ethn_diversity | Fraction of ethnic backgrounds in a grid cell |  |
| Environmental characteristics | residential | Fraction of residential land use in a grid cell | Open data repository Flemish government |
|  | industry | Fraction of industrial land use in a grid cell |  |
|  | commercial | Fraction of commercial land use in a grid cell |  |
|  | services | Fraction of land used for services in a grid cell |  |
|  | transport | Fraction of land used for transportation in a grid cell |  |
|  | recreation | Fraction of recreational land use in a grid cell |  |
|  | other_build | Fraction of build land use in a grid cell |  |
|  | other_unbuild | Fraction of unbuild land use in a grid cell |  |
|  | water | Fraction of land use pertaining to water in a grid cell |  |
|  | green | Fraction of green land use in a grid cell |  |
|  | dens_bank | Density of banks in a grid cell | OpenStreetMap |
|  | dens_gas | Density of gas stations in a grid cell |  |
|  | dens_gym | Density of gyms in a grid cell |  |
|  | dens_hairdresser | Density of hair salons in a grid cell |  |
|  | dens_hotel | Density of hotels in a grid cell |  |
|  | dens_nightclubs | Density of nightclubs and bars in a grid cell |  |
|  | dens_restaurant | Density of restaurants and cafes in a grid cell |  |
|  | dens_shops | Density of (regular) shops in a grid cell |  |
|  | dist_bank | Distance to the nearest bank |  |
|  | dist_gas | Distance to the nearest gas station |  |
|  | dist_gym | Distance to the nearest gym |  |
|  | dist_hairdresser | Distance to the nearest hair salon |  |
|  | dist_hotel | Distance to the nearest hotel |  |
|  | dist_nightclub | Distance to the nearest bar or nightclub |  |
|  | dist_rest | Distance to the nearest restaurant or cafe |  |
|  | dist_shops | Distance to the nearest shop |  |
|  | dist_education | Distance to the nearest educational facility |  |
|  | dist_retail | Distance to the nearest retail shop |  |
|  | dist_firestation | Distance to the nearest fire station |  |
|  | dist_hosp | Distance to the nearest hospital |  |
|  | dist_police | Distance to the nearest police station |  |
|  | dist_library | Distance to the nearest library |  |
|  | dist_publicbuild | Distance to the nearest public building (excluding fire and police stations and hospitals) |  |
|  | dist_train | Distance to the nearest train station |  |
|  | dist_bus | Distance to the nearest bus stop |  |
|  | dist_tram | Distance to the nearest tram stop |  |
|  | dist_highway | Distance to the nearest highway |  |
|  | function_mix | Function mix score | Walkability tool of the Flemish government |
|  | street_connect | Street connectivity score |  |
| Temporal | temp | Mean temperature | OpenWeather |
|  | dew_point | Mean dew point |  |
|  | pressure | Mean air pressure |  |
|  | wind_speed | Mean wind speed |  |
|  | humidity | Mean humidity |  |

#
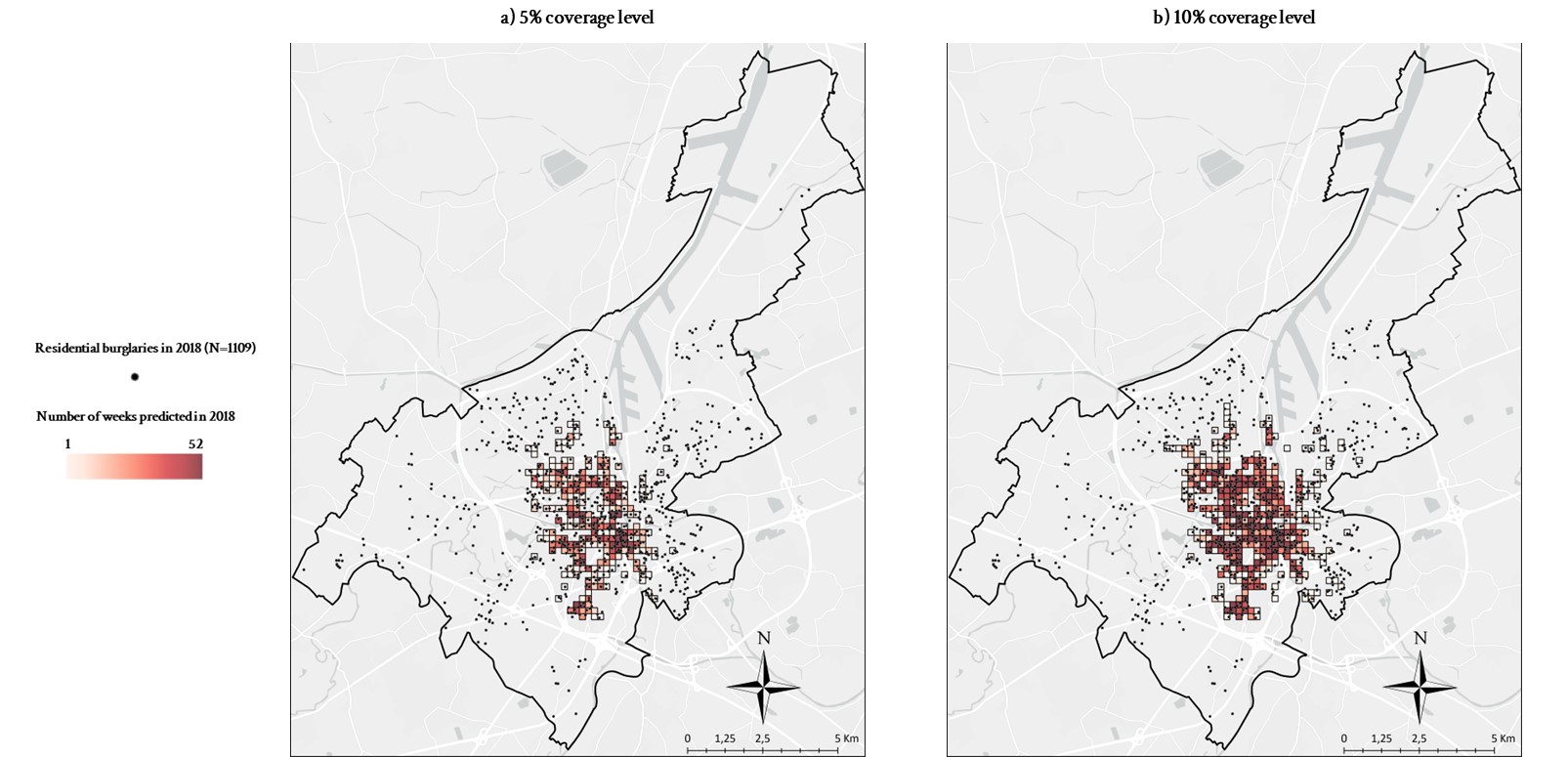
**Appendix B: Spatial visualization of predicted patterns**

# **
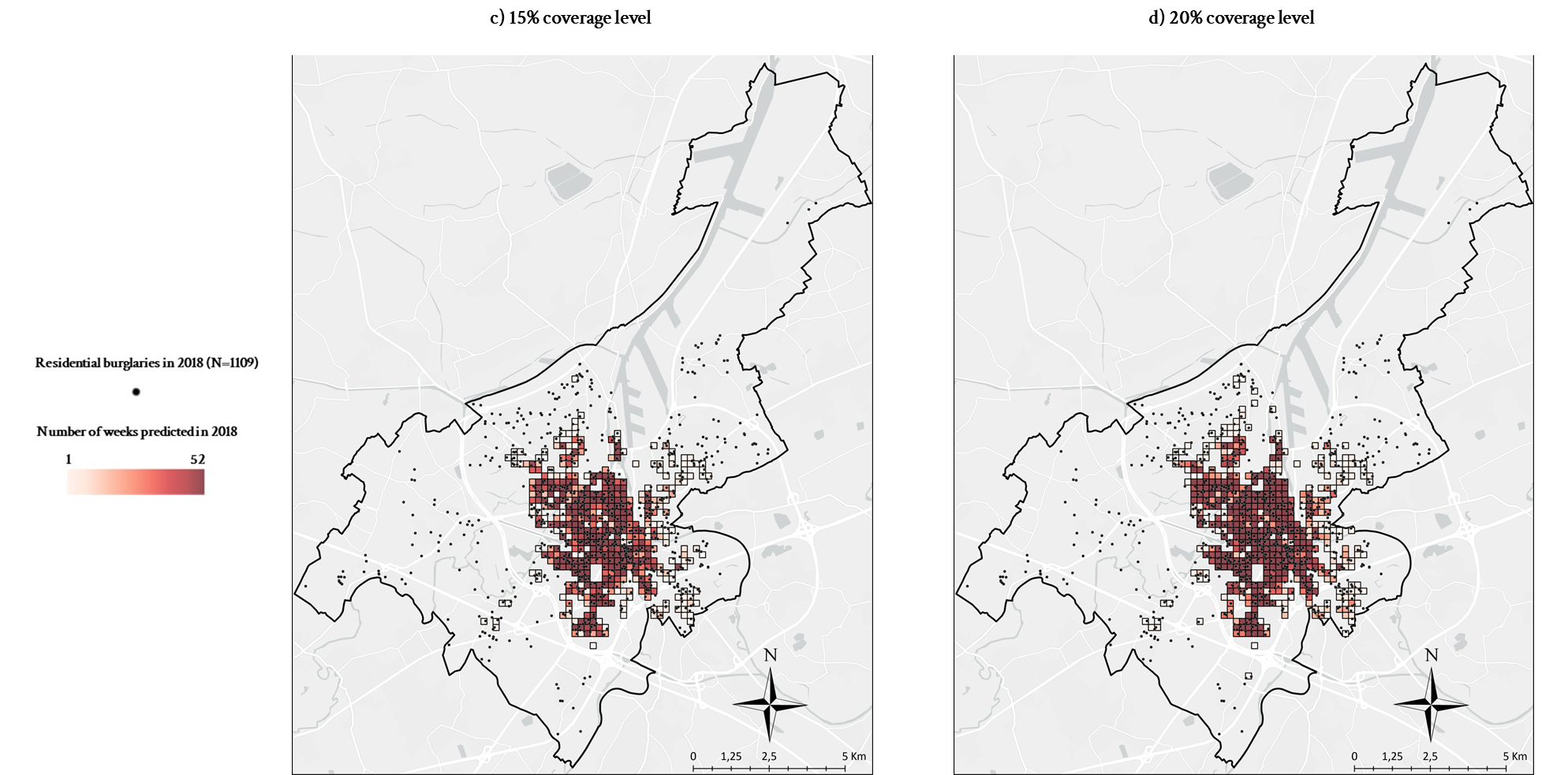
** **Appendix B: Spatial visualization of predicted patterns**
